# Evolution of maternal and zygotic mRNA complements in the early *Drosophila* embryo

**DOI:** 10.1371/journal.pgen.1007838

**Published:** 2018-12-17

**Authors:** Joel Atallah, Susan E. Lott

**Affiliations:** Department of Evolution and Ecology, University of California, Davis, Davis, California, United States of America; University of Georgia, UNITED STATES

## Abstract

The earliest stages of animal development are controlled by maternally deposited mRNA transcripts and proteins. Once the zygote is able to transcribe its own genome, maternal transcripts are degraded, in a tightly regulated process known as the maternal to zygotic transition (MZT). While this process has been well-studied within model species, we have little knowledge of how the pools of maternal and zygotic transcripts evolve. To characterize the evolutionary dynamics and functional constraints on early embryonic expression, we created a transcriptomic dataset for 14 *Drosophila* species spanning over 50 million years of evolution, at developmental stages before and after the MZT, and compared our results with a previously published *Aedes aegypti* developmental time course. We found deep conservation over 250 million years of a core set of genes transcribed only by the zygote. This select group is highly enriched in transcription factors that play critical roles in early development. However, we also identify a surprisingly high level of change in the transcripts represented at both stages over the phylogeny. While mRNA levels of genes with maternally deposited transcripts are more highly conserved than zygotic genes, those maternal transcripts that are completely degraded at the MZT vary dramatically between species. We also show that hundreds of genes have different isoform usage between the maternal and zygotic genomes. Our work suggests that maternal transcript deposition and early zygotic transcription are remarkably dynamic over evolutionary time, despite the widespread conservation of early developmental processes.

## Author summary

Genetic control of embryonic development in all animals requires precise coordination between mother and zygote. The mother provides gene products to the egg to drive the earliest stages of development, until the zygote is able to transcribe its own genome. Many processes of early development are highly conserved over evolutionary time, and are critical for organism survival. Here, we determine how conserved the pools of transcripts provided by the mother and transcribed by the zygote are over evolutionary time, by sequencing all the transcripts at each stage in 14 species of fruit flies at different stages of development. We find a substantial conservation of transcripts represented, with the transcripts deposited by the mother being especially highly conserved. However, we also find considerable variation in these early transcript pools between species, suggesting that while early developmental processes are highly conserved, the gene products driving them may not be. This may be necessary for carrying out the processes of development in different environments.

## Introduction

Most early developmental processes, such as rapid cleavage cycles and the establishment of body axes, are shared across multicellular animals, but the extent to which the mechanisms and the genes involved are also shared remains an open question.

Throughout the animal kingdom, the first stages of development are controlled by mRNA transcripts and proteins deposited by the mother during oogenesis. Genetic control is subsequently transferred from the maternal genome to the zygotic genome. This is accomplished through a precise and elegant series of regulatory steps, in which the zygotic genome is transcriptionally activated while maternal transcripts are degraded, in a process known as the maternal to zygotic transition (MZT; [1,2]. This handoff between mother and zygote has the appearance of a functional logic that dictates which genome is in control. Genes involved in processes unique to the earliest stages of development, such as rapid cleavage cycles, are necessarily transcribed by the mother. Genes that control processes such as patterned gene expression in the developing embryo require zygotic transcription from specific subsets of cells. And genes performing essential housekeeping functions required at all stages of life are transcribed by both the mother and the zygote. For these genes, the maternal and zygotic genomes are able to coordinate during the MZT to such a degree that the transcript levels of these genes can be relatively constant, despite the transition between genomes of origin for these transcripts.

In general, the logic underlying the partitioning of gene expression between the maternal and zygotic genomes is unclear. While we have examples of particular genes that are transcribed by either (or both) the maternal and zygotic genomes, in accordance with the requirements discussed in the previous paragraph, it is unknown whether these requirements play out to shape maternal and zygotic gene expression genome-wide. One way to address this question is to analyze evolution of transcript pools at these stages on short to moderate timescales. If the genome of origin is a constraining factor for many genes, we would expect to see a high degree of conservation of maternal or zygotic expression for those genes. Alternatively, if transcripts of some genes may be supplied by either mother or zygote, we might expect to see control of expression vary between the two genomes across species.

The current evidence for conservation of maternal and early zygotic regulatory factors is mixed. One of the most critical maternal genes in the fruit fly *Drosophila melanogaster*, *bicoid* [3], is of relatively recent evolutionary origin. Bicoid controls axis formation (determines the anterior pole of the egg), and is not found outside of higher Diptera [4], having resulted from a gene duplication. This demonstrates that conserved early developmental processes can incorporate new genes. Some theory and empirical studies suggest that maternal genes might be expected to evolve more quickly than zygotic genes, as selection will be less effective since these are (largely) autosomal genes that are expressed in only one sex [5–7]. These studies examined coding region changes in a limited number of genes, and might not fully account for the significant developmental constraint imposed by the need to build functional offspring. However, recent genomic studies demonstrate a high degree of maternal transcript level conservation relative to zygotic gene expression [8,9].

Mechanistically, maternal deposition and zygotic expression are subject to different constraints. Prior to zygotic genome activation, deposited maternal gene products are the only mRNA transcripts available, and transcript level cannot be dynamically increased to respond to the rapidly changing environment of the earliest developmental stages. Perhaps because of this, post-transcriptional control mechanisms play an especially important role in the levels of gene products produced from maternal transcripts [10–15]. On the other hand, since the products of many zygotic genes are needed soon after transcription is activated, the accumulation of sufficient transcripts in a short period of time (especially in species with rapid development such as *Drosophila*) can be difficult. For this reason, genes expressed in *Drosophila* at the early zygotic stage tend to be short in length and contain few or no introns [9,16,17], allowing them to be transcribed quickly. Since both the maternal and zygotic phases of early development have unique constraints, predicting the level of conservation of each stage is challenging.

In order to assess the extent of transcript level conservation in early development across evolutionary time, we sequenced transcriptomes from early embryonic stages from 14 *Drosophila* species. These species span divergence times of approximately 250,000 to close to 60 million years. We determined mRNA levels both before and after zygotic genome activation, analyzing the conservation and divergence of transcript representation across species over evolutionary time.

Our findings show strong levels of evolutionary conservation of both maternal and zygotic transcripts. However, genes that are represented at only one stage (either before or after zygotic genome activation) show strikingly high degrees of transcript level evolution compared to genes represented at both stages. The results suggest that in most cases robust transcript levels may be achieved through regulatory mechanisms that rely on both the maternal and zygotic genomes. Nevertheless, when all transcripts at the maternal stage are compared to all the genes at the post-genome activation stage, the maternal stage has a higher degree of conservation. This is in contrast to the pattern observed with stage-specific transcripts, where transcripts present at the maternal stage that are entirely degraded at the MZT evolve faster than zygotic genes with no maternal contribution. Furthermore, we find that expression levels of a small proportion of zygotic genes with no maternal contribution are tightly regulated, and the use of stage-specific isoforms may be a hitherto unrecognized method of partitioning the contributions of two genomes. Finally, combining our results with data from the mosquito *Aedes aegypti*, we show the conservation of a core set of zygotic genes over 250 million years. Our study demonstrates the power of transcriptomic phylogenetics to identify the key players regulating core developmental processes.

## Results

In order to characterize how transcript representation and abundance across the maternal to zygotic transition changes over evolutionary time, we have created an RNA-Seq dataset from 14 *Drosophila* species at two developmental timepoints. The 14 species chosen represent divergence times of 0.25–57 million years [18], considerable life history variation [19], and fully sequenced genomes [20–22]. We sampled two developmental timepoints, one (stage 2, Bownes’ stages) where all transcripts are maternally derived, and another (the end of stage 5/blastoderm stage) after the onset of zygotic transcription, and just prior to gastrulation. Since these developmental stages are morphologically distinct [23,24] and highly conserved [25], comparable timepoints can be identified across species. Each species and timepoint was represented by at least 3 single-embryo replicates (S1 Table; see Methods for full description of the dataset), although in one case (*Drosophila ananassae*) only two stage 2 replicates were used in the analysis.

### Reproducibility of the data

**Fig 1:**
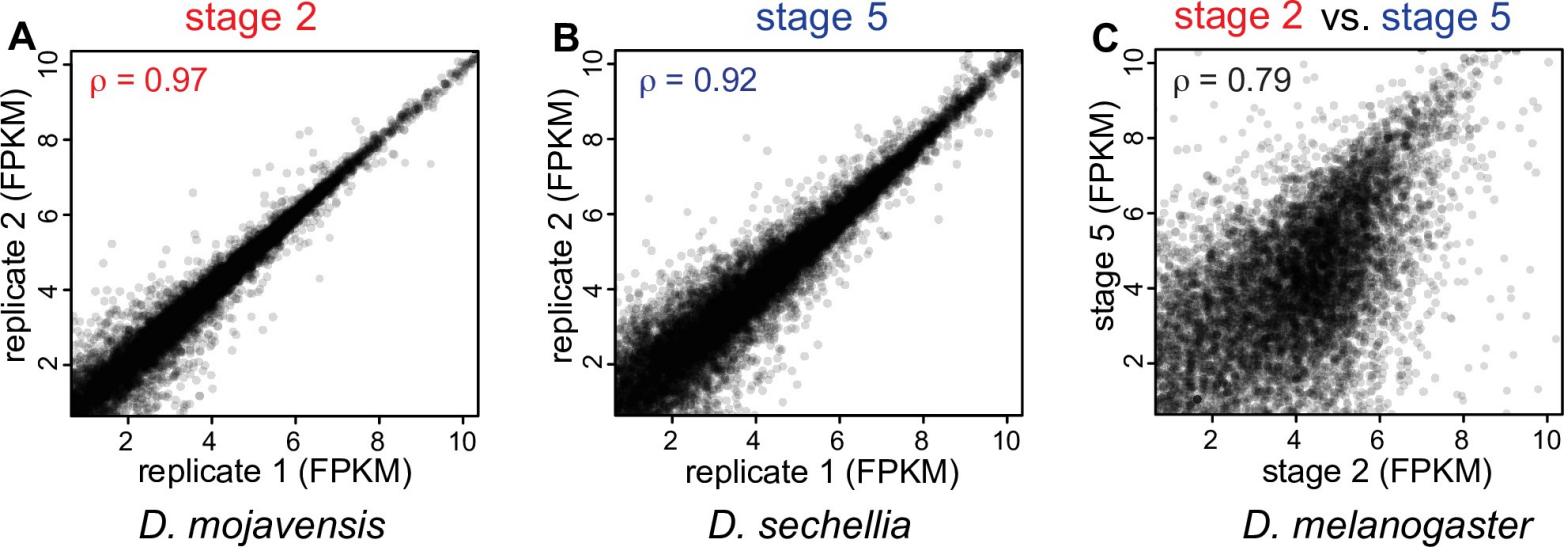
Reproducibility of the data. (A,B) Spearman rank correlation coefficients are high when FPKM values of embryonic transcriptomes at the same stage are compared. (C) The transcriptome changes dramatically between stage 2 and stage 5.

Our results confirm our previous finding that single-embryo RNA-seq is highly reproducible [26–28]. Spearman rank correlation coefficients of transcript abundance (in fragments per kilobase per million reads mapped, or FPKM) for replicates from the same stage are high at both developmental stages (S1 Table), though slightly lower at stage 5 (post-zygotic genome activation) than stage 2 (maternal transcripts only). For stage 2, the correlation coefficients within species range from 0.94–0.98 across 13 of the 14 species (Fig 1A; S1 Table), with a mean of 0.96. *D*. *erecta* stage 2 transcriptomes have slightly lower correlations (0.91–0.96). Spearman coefficients for stage 5 replicates (Fig 1A; S1 Table) are always lower than their stage 2 counterparts and are also more variable, ranging from 0.80 to 0.95, with a mean of 0.90. They are still higher, however, than correlation coefficients of replicates from different stages (for any given species), which range from 0.67 to 0.83, with a mean of 0.76 (Fig 1C; S1 Table).

There are a few reasons why stage 5 transcript levels may be expected to be less correlated than stage 2, even within replicates. First, stage 5 transcript levels are the result of both zygotic transcription and maternal transcripts that have yet to degrade. Approximately 50% of maternal transcripts are still present at this timepoint [1,17,29], and degradation of maternal genes may vary between species [28]. As full activation of the zytogic transcriptome begins during stage 5, small differences in developmental timing between replicates in this stage may also produce differences in zygotic transcripts present. To address this, our stage 5 timepoint is a precise point at the end of this stage, when cellularization has completed, but prior to gastrulation (see Methods). Finally, embryo sex may begin to play a role by the end of stage 5. As the sex determination pathway has been activated by this stage, it is possible to distinguish males from females by comparing levels of known female- and male-specific transcripts (*Sxl* and *msl-2*) [30]. While sex-specific differences in stage 5 transcript abundance across species have been described previously [28], there are no consistent differences between Spearman rank sum comparisons of same-sex vs. opposite-sex stage 5 embryos (S1 Table), suggesting that these differences are overwhelmed by transcript levels from non-X-linked genes when comparing whole transcriptomes.

Since replicate FPKM levels are highly correlated, our analysis in the remainder of this paper focuses on mean replicate FPKM values for a given species and stage.

### Divergence in stage-specific mRNA levels increases gradually over evolutionary time

**Fig 2:**
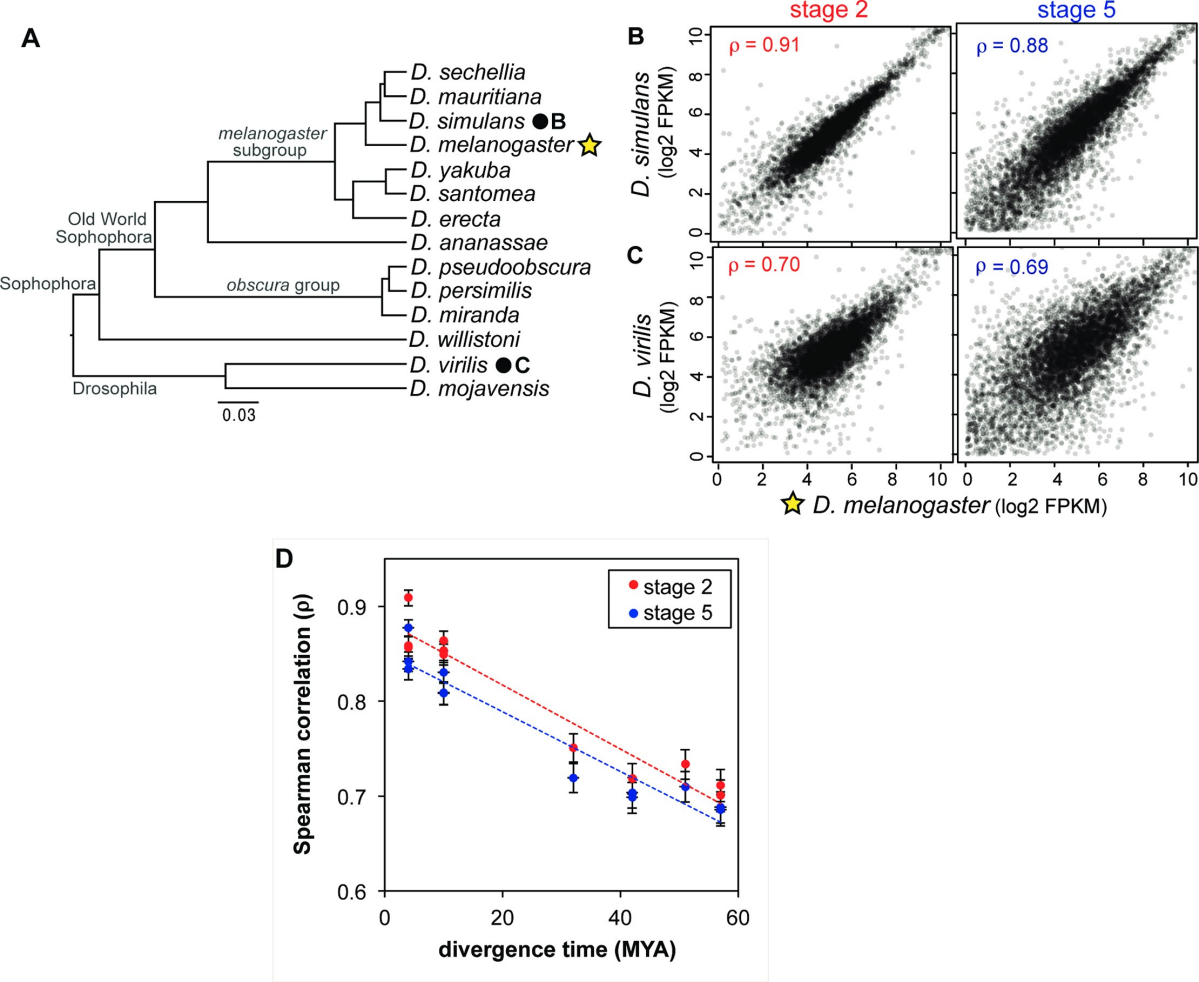
Evolution of the early transcriptome. Stage-specific transcriptomes are highly correlated across species. (A) Phylogeny of species in the study. The star represents the focal species, to which the species with filled circles are compared in parts B and C. (B,C) The *D*. *melanogaster* (star, 2A) stage 2 and stage 5 transcriptomes are more highly correlated with comparable transcriptomes (dots) from the closely related *D*. *simulans* (B) than the distantly related *D*. *virilis* (C). (D) When comparing *D*. *melanogaster* with the other species, Spearman correlation coefficients decrease over evolutionary time.

While transcript levels within each stage are highly correlated across species (Fig 2; S2 Table), they diverge as evolutionary distance increases. Spearman rank correlation coefficients decrease when comparing a species from within the *melanogaster* subgroup (e.g. *D*. *melanogaster*) with more distantly related flies, but only drop to around 0.7 for divergence times of approximately 57 million years (e.g. *D*. *melanogaster* to *D*. *virilis*, Fig 2C). As was found with replicates from the same species, stage 5 correlation coefficients are usually slightly lower than the equivalent stage 2 coefficients (Fig 2B and 2C; S2 Table). In contrast, cross-species comparisons of different stages show striking differences. Clustering the transcriptomes from all species and timepoints (S1 Fig), the two stages fall out as the first two distinct clusters, demonstrating that the biological distinctiveness of these developmental stages in *Drosophila* transcends interspecific variation.

While divergence in transcript levels generally increases with evolutionary distance, transcriptome comparisons do not recapitulate the phylogeny (S1A Fig). In particular, the *obscura* group shows strong transcriptomic divergence in excess of its phylogenetic distance from other species. The *obscura* group has experienced a number of fusions of sex chromosomes, resulting in a larger proportion of the genome being sex-linked [31]. To determine if this could be driving the pattern we observe, we performed the clustering analysis on autosomal genes, removing all gene groups where one or more orthologs were located on a sex chromosome in any of the species with chromosomal-level annotations. This required removing gene groups with an ortholog on the X chromosome, or Muller element A, in *D*. *melanogaster*, *D*. *simulans*, *D*. *mauritiana*, *D*. *yakuba*, *D*. *pseudoobscura*, and *D*. *miranda*, in addition to gene groups with an ortholog on Muller D in *D*. *pseudoobscura* or *D*. *miranda* or an ortholog on Muller C in *D*. *miranda* [32–34]. We found that the *obscura* group still clusters together outside the rest of the species when sex-linked genes were removed from the analysis (S1B and S1C Fig). As this group represents the only clade in our study adapted to a temperate environment (*D*. *virilis* is also considered a temperate species, but has no close relatives in our dataset), some of the differences in this group may be ecologically relevant. We return to this observation below.

### Evolution of stage-restricted transcripts

**Fig 3:**
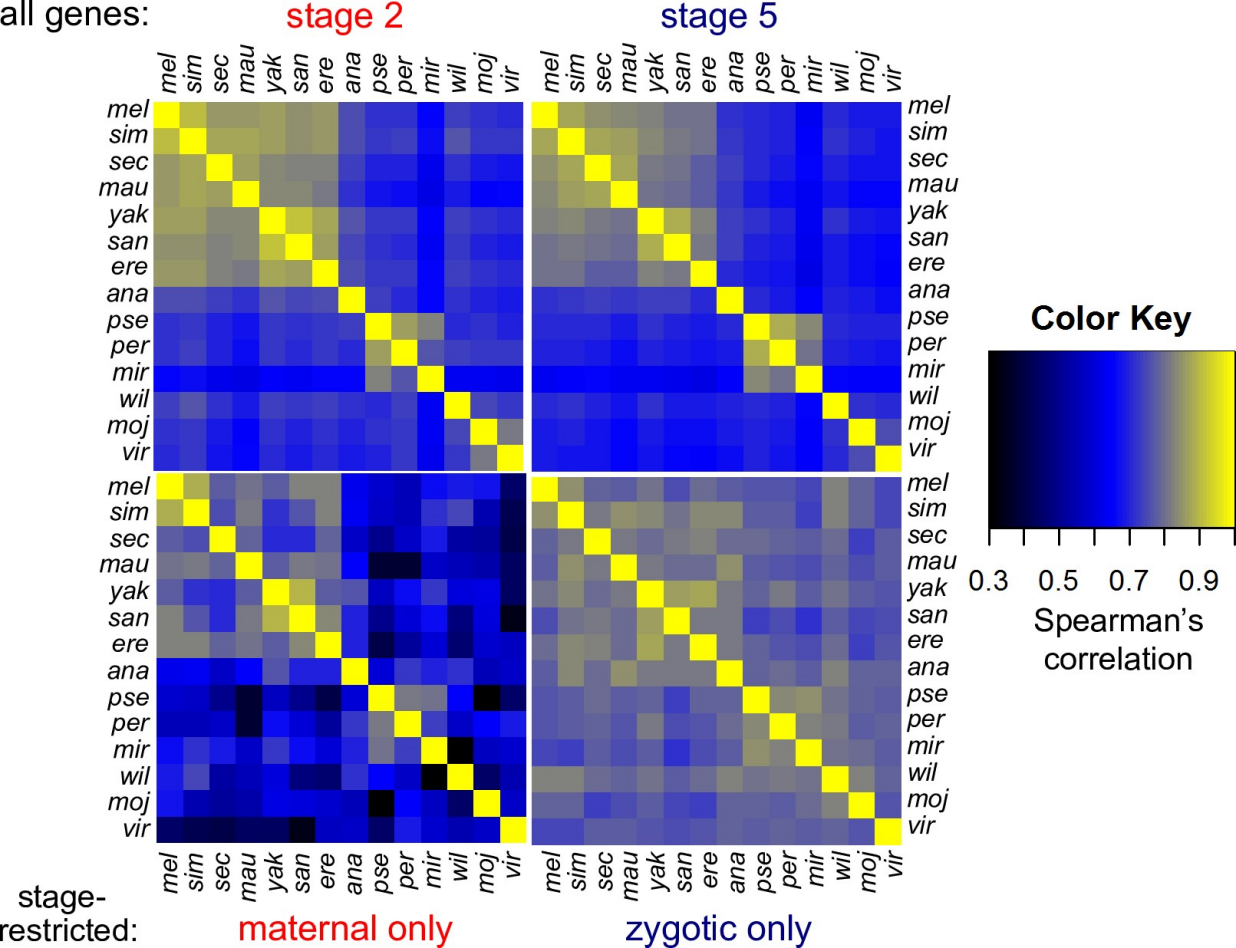
Evolutionary transcriptomic divergence across different classes of genes. Separate correlation plots show interspecific pairwise Spearman correlation coefficients of transcript levels (FPKM) of all genes represented at stage 2 and stage 5 in both species (top row) and genes that are maternal-only (represented at stage 2 and with all transcripts degraded by stage 5) or zygotic-only (represented at stage 5 and not maternally deposited) in both species (bottom row). When comparing species that are closely related (e.g. members of the *melanogaster* subgroup or *obscura* group), Spearman coefficients are relatively high for all stage 2, all stage 5 and maternal-only genes, and drop sharply when more distant species are compared. This pattern is less obvious for genes that are zygotic-only in the two species being compared, which show relatively high correlations at large evolutionary distances.

We then focused on stage-restricted genes, either maternal-only (maternally deposited and entirely degraded by stage 5) or zygotic-only (not maternally deposited, transcribed from the zygotic genome and present at stage 5); see Methods for further definitions. The number of maternal-only genes is relatively small; on average this group represents ~6% of transcripts present at either or both of these stages (S3 Table). In comparison, zygotic-only transcripts represent ~20% of those present at these stages (S3 Table). When comparing species pairs, we found that as evolutionary distance increases, the number of orthologs that are restricted to the same stage in both species declines, particularly in the maternal-only set (S2 Fig). If the transcript levels of genes that are restricted to a given stage in either of the two species are compared, we find that correlation coefficients for both types of stage-restricted genes are markedly lower than those for all stage 2 and stage 5 genes (S3 Fig). However, if we only compare the transcript levels of genes that are stage-restricted in both species (Fig 3), we see a striking contrast between the maternal-only group, which shows strong transcriptomic divergence as evolutionary distance increases, and the zygotic-only group, which shows remarkable conservation among even the most distant species in our analysis. This suggests that while both groups of stage-restricted genes show rapid evolution relative to non-stage restricted genes present at these stages, the transcript levels of a subset of zygotic-only genes are more tightly regulated across *Drosophila* clades.

### A core set of zygotic-only genes are conserved between Drosophila and basal Diptera

To investigate evolution of transcript pools in early development over longer periods of evolutionary time, we incorporated data from the previously published developmental transcriptomic time series of a basal Dipteran, the mosquito *Aedes aegypti*, which separated from *Drosophila* 170 to 250 million years ago [35]. Since *Aedes aegypti* shares the long-germ band mode of development with Drosophila [36], where the body plan is established simultaneously rather than sequentially [37], early developmental processes are largely conserved. In the *Aedes aegypti* dataset, all transcripts are maternal at the earliest stage assayed (0–2 hours; the equivalent of Drosophila stage 2), while the approximate equivalent of *Drosophila* stage 5 is around 10 hours, when *Aedes* cellularization is complete [38], covered by the 8–12 hour window in this time series. We will refer to these stages as *Aedes* stage 2 and *Aedes* stage 5, respectively.

**Fig 4:**
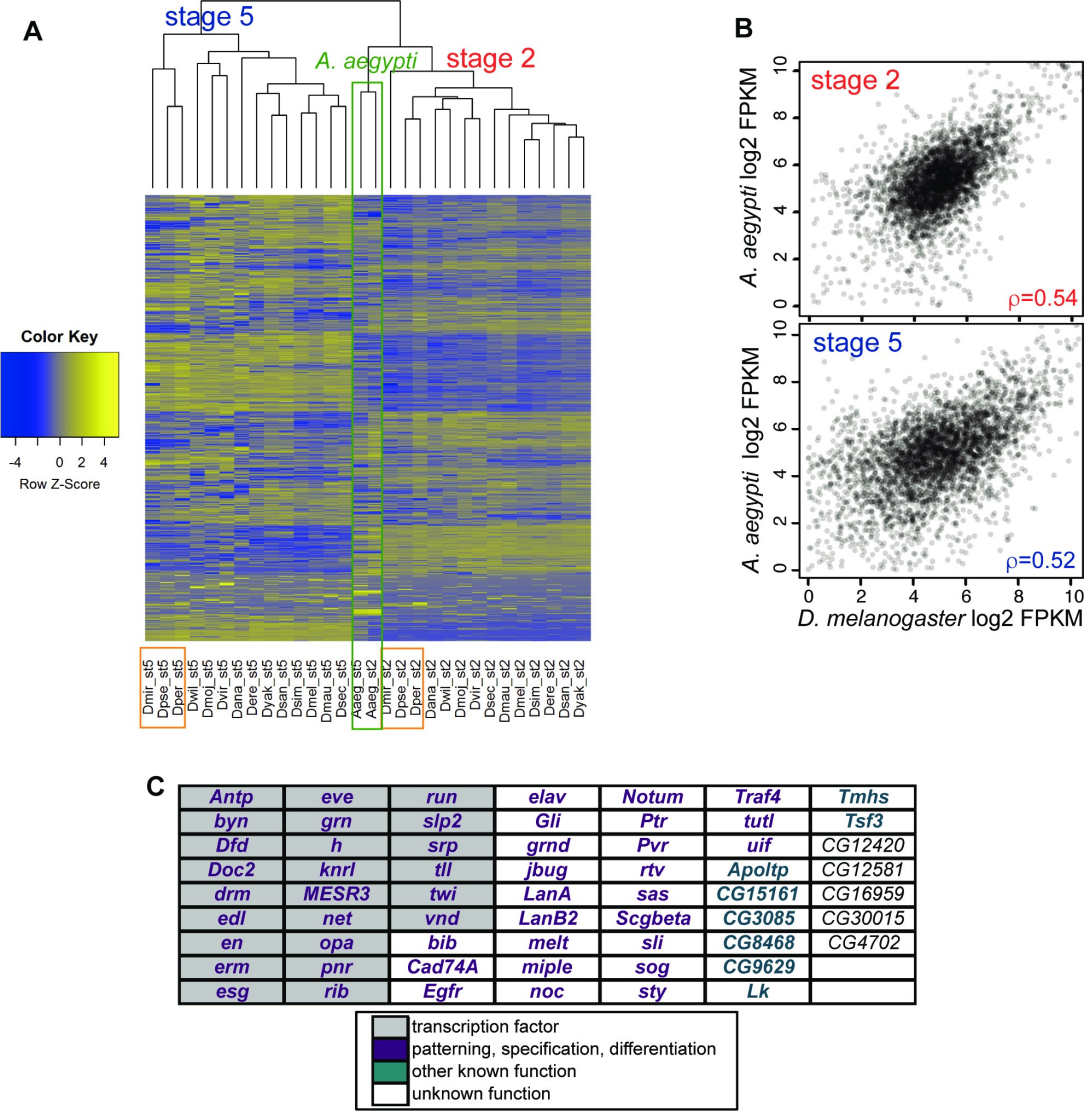
Comparison to *Aedes aegypti* highlights patterns at longer evolutionary divergence times. (A) Transcriptomes of each stage cluster together within *Drosophila*, while the equivalent stages of *Aedes aegypti* (250 MY diverged) cluster by species rather than by stage. Within *Drosophila*, clustering does not fully recapitulate the phylogeny. For both stage 2 and stage 5, the *obscura* group (orange boxes) form an outgroup to those from all other species of an equivalent stage. This is true when all transcripts are examined (this plot) or only autosomal transcripts are examined (S1C Fig). B) Transcript levels (FPKM) for all transcripts at each stage are relatively highly correlated between *Drosophila* (here *D*. *melanogaster*) and *Aedes aegypti*. C) Genes that have conserved zygotic-only representation in both *Drosophila* and *Aedes* are highly enriched in transcription factors, as well as known developmental functions. A small number of these genes have unknown functions in the early embryo.

As shown in hierarchical clustering analysis of the *Drosophila* and *Aedes* transcriptomes (Fig 4A, S1C Fig), the *Aedes* transcriptomes from both developmental stages cluster together, rather than clustering with the comparable *Drosophila* stages, suggesting that evolutionary divergence of this magnitude is more significant than divergence between developmental stages. However, despite this divergence, and in spite of the differences in the experimental protocols that generated the data (the *Drosophila* transcriptomes were generated from single embryos, while the *Aedes* time series used pooled embryos), there are still moderately strong and highly significant correlation coefficients between transcript levels of orthologous genes from the respective stages (Fig 4B, S2 Table). We were particularly interested in analyzing the classes of genes that showed the greatest change in representation within *Drosophila* (maternal-only and zygotic-only) and determining whether we could find conserved orthologs between *Drosophila* and basal Diptera. In doing so, we found considerable change in stage-specific genes at this evolutionary distance, but also a core set of highly conserved zygotic-only genes likely to have important functions.

Considering first the zygotic-only genes, we examined two sets of genes: 1) genes that displayed any evidence of conservation between *Aedes* and a *Drosophila* species, and 2) a subset of these genes that also showed strong evidence of conservation within the *Drosophila* clade. Looking first for any conservation between *Aedes* and *Drosophila*, we found 173 genes (from a set of 4619 orthologs identified across both stages) that were zygotic-only in both *Aedes* and at least one of the 14 *Drosophila* species (S4 Table). This set of 173 genes represents approximately 10% of the mean number of zygotic-only genes in a *Drosophila* species (S3 Table). A given gene in this set was, on average, also zygotic-only in 6.5 other *Drosophila* species (S4A Fig). By contrast, a random gene from the larger dataset of 4619 orthologs was on average zygotic-only in less than one *Drosophila* species. To identify a more stringent set of genes that are also conserved within *Drosophila*, we required genes to be be zygotic-only in *Aedes aegypti* and two of the earliest-diverging *Drosophila* species (*D*. *willistoni*, the outgroup to Old World *Sophophora*, and at least one of the two *Drosophila* subgenus species). With this set of criteria, we identified a core set of 61 genes, which show even greater evolutionary constraint (S4B Fig), and are, on average, zygotic-only in 10 other *Drosophila* species (a total of 13 of the 15 species). This number represents ~4% of the average number of zygotic-only genes in a *Drosophila* species (S3 Table).

Our results indicate that although most zygotic-only genes show rapid change in representation over stages, a core set can be identified where the zygotic-only state is highly conserved (Fig 4C, S5 Table). These 61 genes represent key players in *D*. *melanogaster* embryonic patterning, with gap genes, pair-rule, segment polarity and homeotic genes in the anterior-posterior pathway represented, in addition to numerous genes in the dorsal-ventral patterning pathway. A gene ontology (GO) analysis using DAVID [39,40] comparing this set to the larger set of stage 5-represented genes shows that it is enriched approximately 8-fold in transcription factors (S4C Fig), with terms related to early development (S4D Fig) strongly overrepresented. These results suggest that a naïve analysis based on conservation of transcriptional state can yield remarkable insight into the set of genes that are functionally significant.

In contrast to the zygotic-only genes, we were unable to identify a significant core set of maternal-only (maternally deposited, completely degraded at the MZT) genes that were conserved between *Aedes* and *Drosophila*. Indeed, out of the approximately 4000 orthologs we examined, we only found 3 genes (*beaten path Ia*, *Phospholipase D* and *Sclp*) that were maternal-only in both *D*. *melanogaster* and *A*. *aegypti*. These results, which are consistent with our findings in *Drosophila*, suggest that this subset of the maternal genes (or the maternal-only status) may not be essential for key developmental processes that are conserved across *Diptera*. One possibility is that maternal deposition could be a process with considerable developmental noise, and that degradation might be a method for compensating for non-functional transcripts that are dumped by nurse cells during oogenesis. Alternatively, these transcripts may have clade-specific functions limited to the earliest stage of development.

### A substantial fraction of genes show gain or loss of stage-specific representation within *Drosophila*

**Fig 5:**
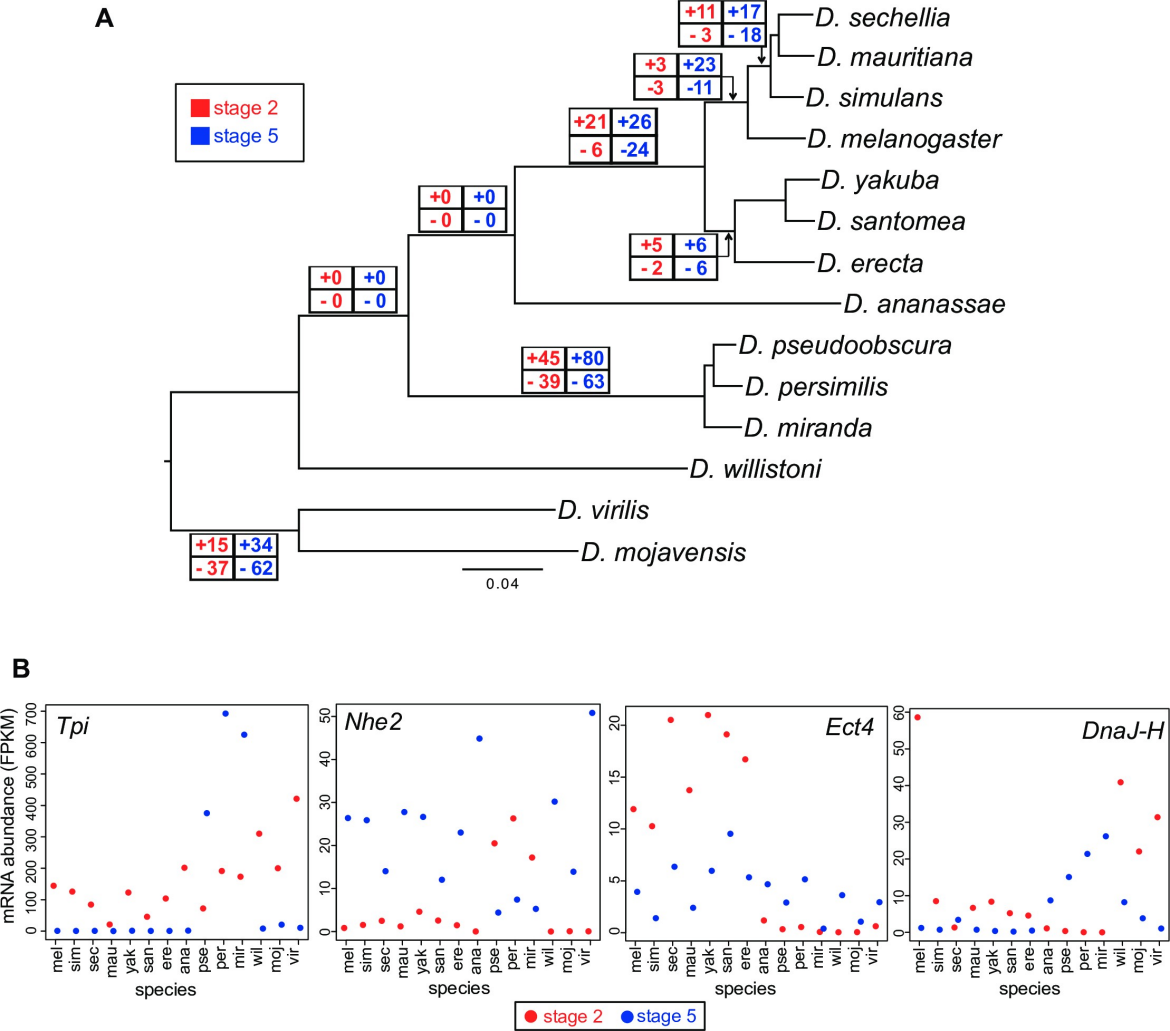
Changes in gene representation across the phylogeny. A) The number of gains and losses of gene representation at each stage are shown for all clades with at least three species. A threshold of FPKM = 1 was used throughout. There are more stage 5 changes (499 transcripts) than stage 2 changes (245), when gradual transitions are included. Only transitions between adjacent nodes are shown. Gains in representation are as common as losses, if not more abundant, across both stages. B) Examples of genes with changes in transcript representation at different stages, in the *obscura* group (first two panels), the *melanogaster* subgroup (third panel), and with highly variable patterns across stages and species (fourth panel).

The previous analysis showed that transcriptomic conservation during early embryonic development is the norm, with RNA levels tightly correlated at up to 60 million years of evolutionary divergence. However, by reconstructing ancestral states (presence or absence of gene transcripts) using the Bayesian phylogenetics package MrBayes [41] (Fig 5; S6 Table), we were also able to identify genes that change in their representation in either the maternal or zygotic transcript pools. For this analysis, a gene was considered represented at a given stage if the FPKM level of its isoforms was at least 1. The ancestral stage 2 and stage 5 states of 7092 genes with one-to-one orthologs in at least 12 of the 14 species were reconstructed (see Methods for more details). Even when using relatively liberal parameters for identifying transitions (see Methods for details), only 245 of these genes, or less than 4%, showed evidence of a stage 2 gain or loss at any node on the phylogeny (Fig 5A). Stage 5 transitions were approximately twice as common, with 499 observed (Fig 5A). By far the greatest number of transitions at both stages were seen in the *obscura* group (see Fig 5B for examples), in support of the hierarchical clustering results (Fig 4A, S1 Fig) suggesting that the early embryonic transcriptome in species of this group is exceptional. An additional finding of note is that loss of representation does not occur with greater frequency than gain of representation (Fig 5A).

Our phylogenetic analysis allowed us to address two major questions: 1) what are the evolutionary patterns of changes in transcript representation relative to maternal, zygotic, or maternal and zygotic representation; and 2) what types of transcripts undergo gains or losses along the tree? To address the former, we compared patterns of gains and losses along the phylogeny. Observing changes in transcript representation at both developmental stages, we rarely find genes that transition from an entirely maternal (maternal-only) state to an entirely zygotic (zygotic-only) state (S7 Table). Indeed, the only gene in S7 Table to show a simultaneous gain of representation at both stages is *quasimodo* (*qsm*), and there is no gene that shows a simultaneous loss at both stages. Primarily, we observe transcripts that are present at both stages losing representation at one stage, or transcripts that are present at one stage gaining representation at the other. This means, for example, that to transition from maternal-only to zygotic-only, the gene would pass through an evolutionarily intermediate state where it is represented at both stages.

Next, we examined which classes of genes change in transcript representation at either stage. We performed gene ontology analysis (see Methods) of genes that change transcript representation when compared with all transcripts present at that stage. In the case of genes that gain stage 5 representation, there is significant enrichment for genes that are involved in transport of small molecules through membranes (ion channel activity, substrate-specific channel activity, ion transport, ion channel complex, channel activity, etc.; see S5 Fig, S8 Table). We might expect transcription of these genes to evolve quickly [42], as their products permit adaptation to new environments on a cellular level. Additionally, some types of these transport mechanisms have been implicated in genetic conflict [43,44] and could thus be expected to evolve quickly. The other categories of transcript abundance changes (stage 5 losses, stage 2 gains, stage 2 losses) had no significant enrichment of gene ontology categories compared to the rest of the genes with transcripts present at that stage.

We highlight a few examples of individual gains of transcript representation in Fig 5B. These particular genes were chosen because they are dramatic examples of changes in transcript representation at different stages and in different species. *Tpi* (*Triose phosphate isomerase*) and *Nhe2* (*Na*^*+*^*/H*^*+*^ *hydrogen exchanger 2*), show gains at stage 5 and stage 2 respectively, in the *obscura* lineage. *Tpi* functions in glycolysis, and is a classic example of clinal allele frequencies in *D*. *melanogaster* populations [45]. *Nhe2* is a Na^+^/H^+^ exchanger that increases cellular pH, and has been shown to be upregulated in cold-acclimated *D*. *melanogaster* [46]. Consistent with this previous evidence of a role in environmental adaptation, both genes showed gains in our cold adapted (temperate) *obscura* group species.

In most cases, however, it is difficult to interpret the significance of the change in representation. *Ect4 (Ectoderm-expressed 4*, Fig 5, third panel), was one of number of genes that showed gains of expression in the *melanogaster* group. In this case, there was a gain in stage 2 representation in this lineage, while stage 5 was also represented in the outgroup. The role of *Ect4* has largely been determined relative to its function in axon degeneration for the purposes of repair after injury, which is not directly relevant to its role in the early embryo. Recently, *Ect4* was shown to be a target of DPP signaling in the embryo [47], but its precise function is unknown. We also found genes, such as *DNAJ-H*, that showed highly variable transcript levels across the phylogeny. Transcripts of this gene, which is part of the Heat Shock Protein 40 family of co-chaperones (essential factors in the Hsp70 chaperone cycle), may be represented at both stages or at either of the two stages, with no apparent pattern relative to the phylogeny.

These changes provide evidence that the early embryonic transcriptome is evolutionarily dynamic within Drosophilids. While the mRNA levels of some genes with key roles in development are highly conserved, a substantial subset of genes whose functions are not as well-understood show evidence of lineage-specific changes that are potentially adaptive.

### Uniquely represented orthologs and unannotated genes

Our results from previous sections suggest relatively rapid evolution of both maternal-only and zygotic-only transcripts (S3 Fig). Transcript levels of a subset of genes where the zygotic-only state is conserved across *Drosophila* species, however, are highly correlated (Fig 3), and a core set of zygotic-only genes with critical developmental roles are conserved with basal Diptera (Fig 4). To extend our analysis, we looked at two special situations: cases of species-specific representation in the early embryo, and the transcriptional profile of a small group of genes that were previously unannotated.

We were first interested in knowing whether there were instances of unique representation: cases where transcripts of a gene were present at a given stage in a species, while transcripts of one-to-one orthologs from all other species assayed were absent from that stage (S9 Table). We only considered genes with one-to-one orthologs in at least 12 of the 14 species. In order to eliminate cases were the transcript level of a gene happened to be slightly above our threshold of 1 in a single species, we focused on instances where a gene had a transcript level over three times the threshold (FPKM > 3) in a single species but an FPKM of less than 1 in all other *Drosophila* species (S6 Fig; S9 Table). We found many more cases of species-specific representation at the stage 2 than stage 5, for every species except *D*. *melanogaster* (which has low numbers of both). There are an exceptionally high number of cases of unique representation of maternal deposition in both *D*. *virilis* and *D*. *erecta*, while *D*. *virilis* also has an unusually high number of instances of unique representation at the zygotic stage (over twice as many as the next highest species, see S9 Table).

We also examined a different situation: unannotated genes identified during analysis by Cufflinks. These genes were not found in the reference annotations (see Methods for annotations used for each species) provided to the software. The number of unannotated genes ranged from a low of 280 in *D*. *melanogaster*, the most thoroughly annotated genome, to a high of 1905 in *D*. *mauritiana* (S10 Table). In many cases, unannotated genes may simply reflect the limitations of previously generated annotations. However, as has previously been found for *de novo* genes [48], unannotated non-*D*. *melanogaster* genes show low complexity, having significantly fewer exons (2–3 vs. the mean from all genes of 4.5–5.5; Wilcoxon rank-sum test, p = 7.4 x 10^−6^), as well as shorter exons (mean of 1240bp vs 2547bp, Wilcoxon test, p = 1.9 x 10^−5^) and introns (mean of 2105bp vs. 5256bp, Wilcoxon test, p = 5 x 10^−8^) than annotated genes (S7 Fig; S10 Table). It is possible, therefore, that some of the unannotated genes identified here are *de novo* genes. We note that these properties of *de novo* genes are shared with annotated zygotic genes, which also tend to have shorter and fewer exons and few or no introns [9,16,17]. The unnanotated genes expressed by the zygotic genome identified here, and reported in the statistics above, are less complex as compared to annotated zygotic genes (S7 Fig; S10 Table).

**Fig 6:**
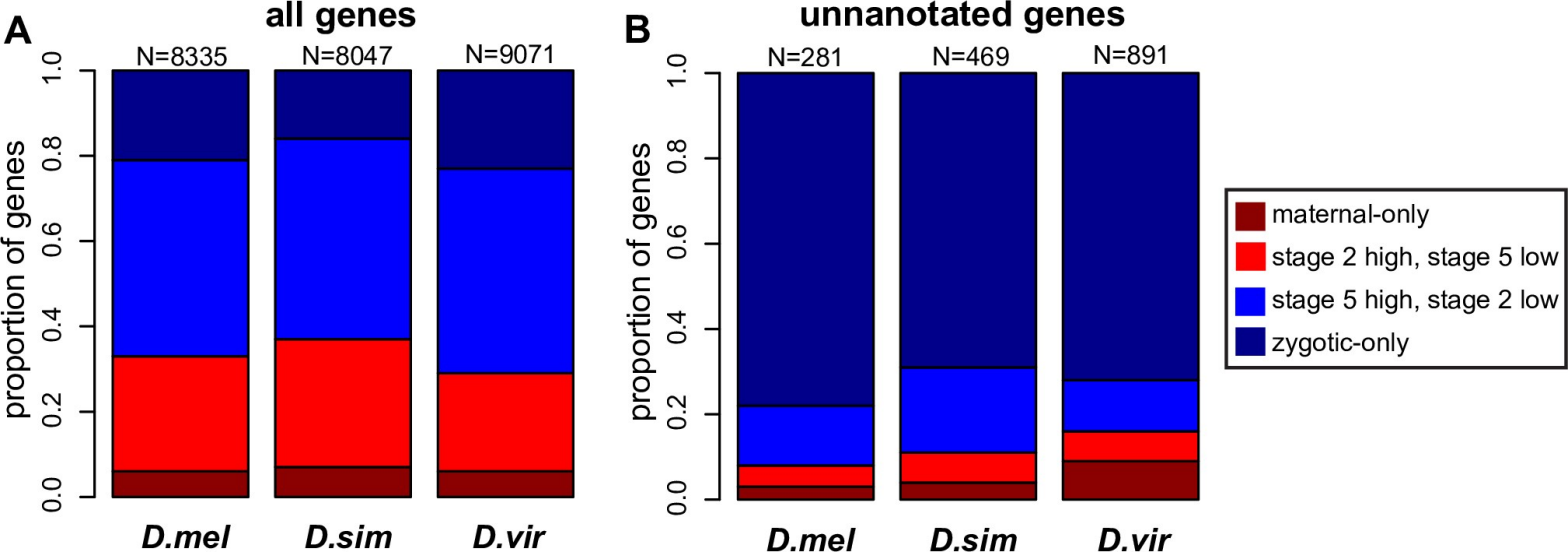
Zygotic-only expression evolves rapidly in unannotated genes. (A) Abundance of genes in different categories (maternal-only, predominantly stage 2, predominantly stage 5, zygotic-only) across species. In addition to the model species *D*. *melanogaster*, *D*. *simulans* is shown as it has the largest proportion of its transcripts represented at stage 2, whereas *D*. *virilis* was chosen as it has one of the largest proportions of zygotic transcripts (*D*. *miranda* was equally as zygotically biased). B) The vast majority of unannotated genes, most of which are taxonomically restricted, are zygotic-only, suggesting that zygotic expression evolves more rapidly than maternal deposition. N indicates the number of genes in each category (all genes, unannotated genes) for each species. See S10 Table for data on all species.

We found that unannotated genes were strongly biased towards being zygotic-only (Fig 6). When considering all genes that are transcribed during this early period of development, only 16–25% are zygotic-only, depending on the species (Fig 6, “all genes”). For the unannotated genes represented by transcripts in our dataset, 65–80% are zygotic-only. This is a 3- to 4-fold increase that is highly significant using a Fisher exact test (p<0.0001). The finding that these unannotated genes are more frequently zygotically transcribed than maternally deposited could indicate that early zygotic transcription evolves rapidly for putatively novel genes. This contrasts with our results for conserved genes (S6 Fig), discussed above, which were found to be more likely to evolve species-specific cases of maternal deposition than zygotic representation. In other words, a potentially novel gene of unknown function will often evolve early zygotic transcription, while a gene with conserved orthologs is more likely to evolve a unique instance of maternal representation.

### Many genes have stage-specific isoforms

The generation of multiple isoforms for a given gene, through mechanisms such as alternate promoters or alternative splicing, has been recognized as a critical form of genetic regulation [49]. Organisms deploy these isoforms in a context-dependent manner to meet the varied challenges of both development and adult physiology. Little is known, however, about the role of alternative isoforms in the maternal to zygotic transition.

In lineages such as *Drosophila*, flies that are adapted for rapid development, it has previously been shown that zygotic transcripts are shorter and have fewer introns than maternally deposited ones [9,16,17]. It is reasonable, therefore, to hypothesize that certain isoforms of the same gene may be better suited for maternal deposition or zygotic transcription. Specific examples of such cases are limited, however, and few studies have looked at the extent to which stage-specific isoforms are evolutionarily conserved.

**Fig 7:**
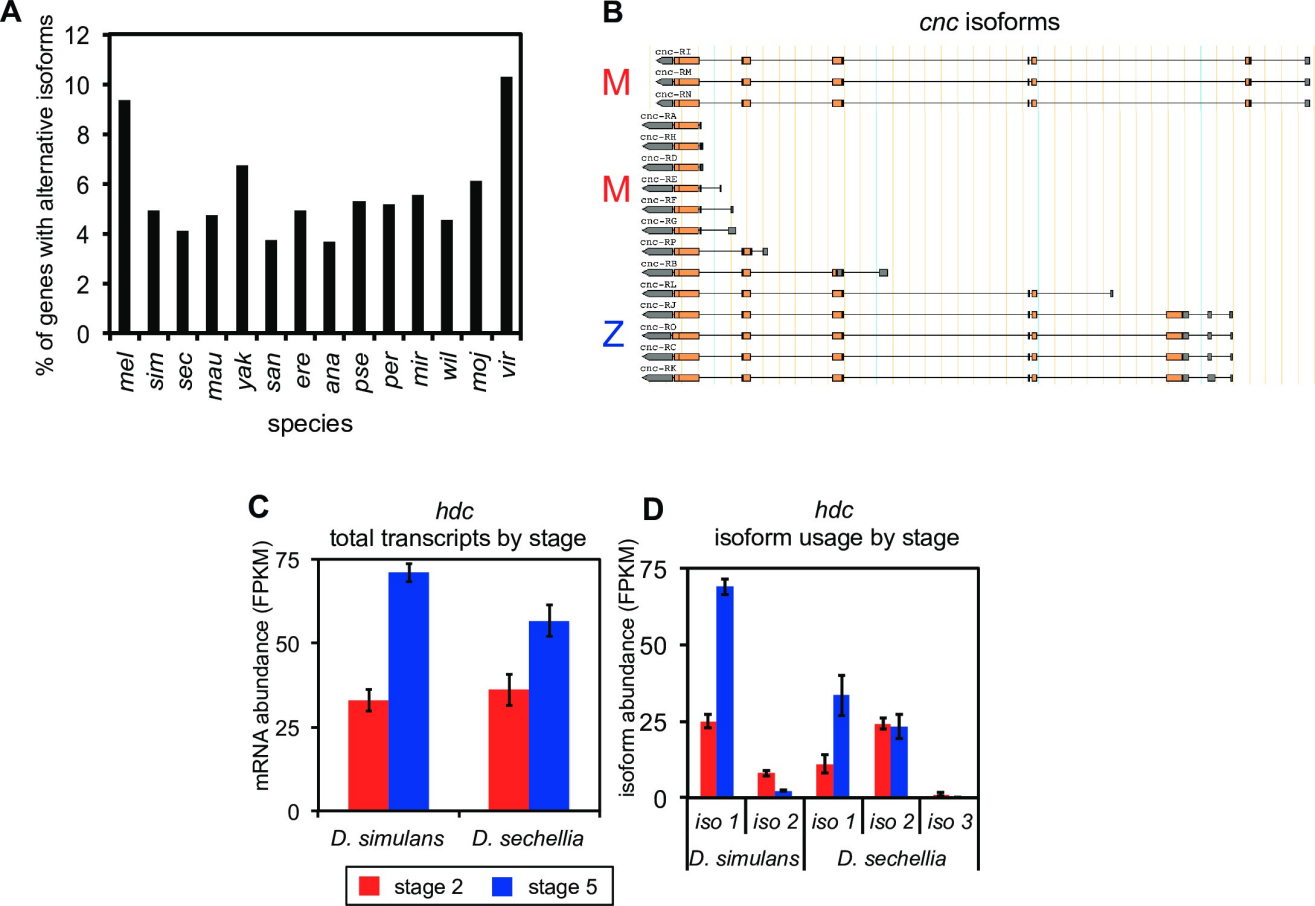
Hundreds of genes have stage-specific isoforms for both stage 2 and stage 5. (A) Genes with separate isoforms for stage 2 and stage 5 (ALT), are found in all 14 *Drosophila* species. (B) Both the long and short isoforms of *cnc* are predominantly stage 2, indicated with the red letter M, while the intermediate length isoforms are predominantly stage 5, indicated with the blue letter Z. While total levels of the *hdc* transcript (C) are similar between sister species *D*. *simulans* and *D*. *sechellia*, the isoform usage evolves even over this short evolutionary timescale (D).

Our data suggest that different isoforms may be used stage-specifically, presenting an additional layer of subtlety when comparing early embryonic transcriptomes across species. Consider, for example, the gene *headcase* (*hdc*), whose function has been characterized at later developmental stages, is expressed in all imaginal lineages, and is involved in trachea, head, and neuroblast development [50]. In the early embryo, transcript levels of orthologs of this gene are present at a higher level at stage 5 than stage 2 in both *D*. *simulans* and *D*. *sechellia* (Fig 7C). However, while both species show zygotic enrichment for the predominant isoform of its ortholog (Fig 7D, labeled “isoform 1” on the figure in both cases), the second most highly expressed isoform of the *D*. *simulans* ortholog (labeled “isoform 2”) is higher at the maternal stage, while the second most highly expressed *D*. *sechellia* isoform shows no significant difference in transcript levels between stages. A third isoform is also present at low levels in *D*. *sechellia*.

To look more closely at the isoforms that are present at each stage, we classified each isoform into one of six categories: maternal (M–only present at stage 2), zygotic (Z–only present at stage 5), predominantly maternal (PM–present at both stages, but at least twice as high at stage 2 than stage 5, with the differences being statistically significant), predominantly zygotic (PZ–present at both stages, but at least twice as high at stage 5 than stage 2, with the differences being statistically significant), maternal-zygotic (present at both stages, but predominant at neither) and not present at either stage (N). When looking across all isoforms, we find that in all species the mean exon number and the mean exonic length are slightly greater for the combined class of M and PM isoforms than for Z and PZ isoforms (S10 Table). The mean intronic length showed much starker differences, and was from 1.5 to 2-fold greater in the M/PM isoforms. This extends the findings from previous studies that zygotic genes are shorter and have fewer introns [9,16,17] to across the genus *Drosophila*, but our data shows much stronger support for differences in intron length than exon number or length between maternal and zygotic transcripts.

We looked specifically for genes that had at least one isoform that was maternal or predominantly maternal, and another that was zygotic or predominantly zygotic, and referred to these as the “alternative” (ALT) set (S10 and S11 Tables). The number of identified ALT genes varied from a low of 307 (~4% of genes) in *D*. *ananassae* to a high of 936 (~ 10% of genes) in *D*. *virilis* (Fig 7A, S10 Table), likely partially a consequence of statistical power. Regardless, the identification of hundreds of genes with stage-specific isoforms (Fig 7A) suggests that this could be an important regulatory strategy in early embryonic development.

In contrast to the patterns we observe for all genes, the ALT isoforms do not differ consistently in exonic number and exonic length (S8 Fig). Across isoforms, the number of exons and exonic length are always greater for isoforms of ALT genes than for those of all genes (S10 Table). These results may indicate that ALT gene products are required to be long and potentially complex to produce stage-specific isoforms, and that a lower proportion of intronic/exonic sequence may be selectively favored in isoforms that are zygotically transcribed.

To explore the functions of these genes with stage-specific or stage-enriched isoforms, we performed a GO analysis. When compared to the entire gene set, we found that these genes are moderately enriched in GO categories of genes that regulate development, morphogenesis and cell differentiation, in addition to sexual reproduction and gamete generation, among several other categories (S12 Table). This is consistent with the fact that one of the most celebrated cases of alternative splicing is in *Drosophila* sex determination, and other the key players in the pathway (*Sex-lethal*, *transformer*, *doublesex*, *fruitless*) being regulated through alternative splicing.

Regardless of their precise functions, the ALT gene state is frequently conserved over long evolutionary distances (S11 Table; S13 Table), with 67 genes showing stage-specific alternative isoforms in orthologs of at least 2/3 of the species. As one example (Fig 7B), consider the case of *cap-n-collar* (*cnc*). This transcription factor, which is essential for viability in *D*. *melanogaster*, has one or more long isoforms (11 exons, 6500–7000 bp of exonic sequence in *D*. *melanogaster*), one or more medium-length isoforms (5 or 7 exons, approximately 4000 or 5000 bp in *D*. *melanogaster*) and one or more short isoforms (3 exons, 3500 to 4000 bp in *D*. *melanogaster*). One-to-one orthologs were identified for 12/14 species (S11 Table), and all of these orthologs except *D*. *mauritiana* were classified as ALT, with the same pattern of at least one long maternal-only, one medium-length zygotic-only, and one short maternal-only isoform found in each case except *D*. *miranda*. The broad conservation of this pattern across 12 species spanning over 55 million years of evolution provides strong evidence for a functional role for both the multiple isoforms and their maternal or zygotic transcription.

## Discussion

### Maternal and post-zygotic genome activation RNA transcript levels are highly conserved

The strong conservation of maternal transcript levels across *Drosophila* demonstrates that the mother’s vast RNA endowment is regulated with a precision that has withstood sixty million years of evolution. This conservation is all the more remarkable given the divergent ecologies and life histories of the species analyzed [19], and the extensive role played by post-transcriptional regulation of maternal transcripts [10–15]. Additional study will be necessary to determine protein abundance in each species, as post-transcriptional regulation may play an additional role in buffering the effects of differential transcript abundance. Since only a small minority of these transcripts are transcription factors, however, it is clear that the function of maternal deposition extends far beyond jumpstarting transcription in development. Theoretical comparisons of selective efficacy notwithstanding [7], maternal genes such as *bcd* that are recent evolutionary innovations [51,52] should be considered the exception rather than the rule.

From another perspective, however, the conservation of stage 5 transcript levels, while somewhat reduced relative to levels of maternal deposition (stage 2), is arguably even more remarkable. Transcript levels at stage 5 are a function of multiple processes: maternal RNA deposition that occurred during oogenesis, multiple embryonic degradation pathways, which themselves may be activated either maternally or zygotically [53,54], and early zygotic transcription. These processes must be tightly coordinated to generate zygotic levels that are reproducible not only between individuals but also between species, a finding that is all the more impressive given the fact that they are regulated in two separate genomes (that of the mother and the zygote).

### Genes that are only represented at one stage show relatively rapid evolution

Transcript levels of two categories of genes show more rapid divergence (S3 Fig): Maternal genes with transcripts that are entirely degraded by stage 5 (maternal-only), and zygotic genes with no maternal contribution (zygotic-only). These results support the hypothesis that the combination of maternal deposition and zygotic transcription is important for achieving the robust transcript levels that might be generally required for early embryonic development. However, when conducting pairwise comparisons in which only the set of genes where the specific stage-restricted state (maternal-only or zygotic-only) is conserved in both species is considered (Fig 3), there are distinct differences in the evolutionary trajectories of these two gene classes.

Maternal-only genes, those maternal genes that are degraded at the MZT and absent by stage 5 evolve quickly. Interspecific correlation coefficients for transcript levels of maternal-only genes drop off rapidly with evolutionary distance, even when only the genes where the maternal-only state is conserved in both species are considered (Fig 3). The number of shared maternal-only orthologs also decreases rapidly with evolutionary distance (S2 Fig), to the extent that there only three *D*. *melanogaster–Aedes aegypti* orthologs that are maternal-only in both clades. How we view these results depends on our interpretation of maternal-only genes. If both the deposition and the degradation are presumed to be functional, these genes would be cases where the transcripts are necessary in the very early, syncytial, embryo (or previously in oogenesis) but are strongly detrimental after cellularization. The finding that an ortholog of such a gene is not maternal-only in a related species would signify that either the gene was no longer necessary in the syncytium or that it was no longer detrimental at later stages. However, our results showing rapid divergence of transcript levels in cases where orthologs are maternal-only in both species, implies that if they have an early function they belong to a class of genes where tight regulation of transcript level may not be necessary. This would distinguish them from the vast majority of genes represented at stage 2, where transcript levels are highly conserved. Alternatively, maternal-only genes may represent developmental noise, with degradation as a method of compensating for the noise. During oogenesis, vast numbers of transcripts and proteins are deposited by the nurse cells in the egg, and it is possible that not all of them are necessary or beneficial to the embryo. Finally, we must consider that the post-transcriptional regulation of maternal transcripts might have a larger impact on the maternal-only transcripts, and that translational control may buffer against any phenotypic consequences of differences in transcript level. This would explain the variation in transcript levels for these genes, but not how quickly the maternal-only status is gained or lost. The sharp decrease in shared orthologs of maternal-only genes as evolutionary distance increases lends weight to the interpretation that maternal deposition may be noisy, and argues against post-transcriptional regulation having a larger role for the maternal-only class of genes as discussed above.

### Complex evolution of zygotic-only genes

The transcript levels of most genes that are zygotic-only diverge rapidly in pairwise comparisons of interspecific orthologs (S3 Fig). However, if we limit our analysis to the smaller group of shared zygotic-only orthologs (genes that are zygotic-only in both species being compared; S2 Fig), we see a very different pattern (Fig 3), with transcript levels highly correlated, even among distantly related *Drosophila* species. Looking across much greater evolutionary distances, we were able to identify a core set of genes that are zygotic-only in both *Aedes* and early-diverging *Drosophila* species. These genes are strongly biased towards retaining the zygotic-only state across the *Drosophila* lineage. Our finding that this set is highly enriched in transcription factors with known functions in embryogenesis shows the power of evolutionary transcriptomics to identify key players in development. Functionally, we might expect these genes to be cases where zygotic-only expression is necessary since maternal deposition may be mechanistically deleterious prior to cellularization.

Across species, an overwhelming majority of unannotated genes have zygotic-only transcript representation in the early embryo, while only about a fifth to a quarter are zygotic-only in the annotated set. Many of the unannotated genes we identified display the hallmarks of newly-evolved genes, with relatively few isoforms and low expression levels, possibly suggesting that novel genes are biased towards zygotic expression (more work will need to be carried out on this gene set to determine whether the genes are indeed taxonomically-restricted). The idea that zygotic representation, on the whole, evolves more readily than maternal deposition is also consistent with our phylogenetic analysis, where a strong majority of the gains were stage 5. The maternal-only genes discussed in the previous section are an exception, as they consist of the minority of maternal transcripts that are entirely degraded by stage 5.

Conversely, maternally deposited RNA differs from its zygotically transcribed counterpart in that it can be used during the earliest syncytial stages of embryonic development. If these early stages are highly conserved, the evolution of new genes with a function in this period may rarely be necessary. Maternal deposition may instead evolve to increase the overall robustness of RNA levels during post-syncytial development. Or, perhaps the earliest stages of development where maternal mRNAs act require different gene products to undergo the conserved developmental processes in differing environments [55].

### Regulation and evolution across the phylogeny

The phylogenetic pattern of evolution of transcript representation at the maternal and zygotic stages speaks to both a regulatory logic and to the relative roles of maternal and zygotic genomes in early development. We found that maternal-only genes hardly ever become zygotic-only (or *vice versa*) between closely related species. Instead, we see genes transcribed by both the maternal and zygotic genomes losing either maternal deposition or zygotic transcription, or stage-restricted genes gaining transcript representation at the other stage (e.g. a maternal-only gene gains zygotic transcription as well). This pattern can potentially be explained by the logic of regulation, since gaining or losing regulatory binding sites (or regulatory factors) at one stage may be a much more common occurrence than simultaneous evolution to both gain one set of binding sites or factors associated with transcription at one stage and lose binding sites or factors for the other stage. At the same time this provides evidence against compensatory evolution over these two stages as loss at one stage is not associated with gain at the other.

### Numerous cases of stage-specific isoforms with conserved structures

While gene number does not appear to correlate with any measure of organismal complexity, it is often claimed that isoform number might [49,56,57]. Alternative splicing is particularly common in vertebrates, although the record for isoform number is currently held by the *Drosophila Dscam1* gene, where isoforms vary across individual neurons [58]. *Drosophila* also famously uses alternative splicing in sex determination [59].

In addition to tissue-specific and sex-specific alternative isoforms, stage-specific isoforms allow for complex temporal regulation [60]. In the early embryo, where both the mother and zygote provide RNA, the logic of utilizing alternative isoforms stems from the differing constraints of each of these players. For example, the rapidity of transcription is a strong selective pressure in the zygote where cell divisions are extremely rapid, leading to zygotic transcripts with fewer introns [16], while maternally supplied RNA is under no such constraint. Additionally, there are transcripts provided by both maternal and zygotic transcription where the maternal transcripts are to be selectively degraded at the MZT. Then the maternal genome could use isoforms with appropriate motifs to direct degradation in their untranslated regions (e.g. miRNA target sites), and the zygotic isoform, without these motifs, will persist.

Our discovery of hundreds of cases of alternative stage-specific isoforms (ASIs) in the 14 species we examined validates the potential utility for using different isoforms at these different stages. Furthermore, multiple cases of strong conservation of isoform structure (number of exons, overall exonic length) for stage 2 or stage 5 isoforms across 60 million years of evolution suggests functionality for this process. Future research will aim to determine if the localization of transcripts differs between the maternally and zygotically predominant isoforms, how these isoforms are differentially regulated, and whether they are functionally equivalent.

### Conclusion

We have demonstrated the remarkable ability of two genomes to collaborate in the regulation of early development, leading to RNA transcript levels in the embryo that are highly stable over tens of millions of years of evolution. Yet, we also find considerable variation in the transcripts present during the earliest stages of development, despite expectations that early development is highly conserved. A large remaining question is how much of this variation is functional. It is plausible that some fraction of it is, and this would imply either that the processes of early development are not as conserved as commonly regarded, or that different complements of transcripts are necessary across different environments and genomes to maintain these conserved early developmental processes. Alternatively, it could be that the processes of early development are remarkably robust, and that considerable variation in transcript abundance or transcript representation may have minimal phenotypic consequences.

## Methods

### Sample collection, library construction, sequencing

Single embryos were collected from 3–8 day old females of each species. Genome lines from the original 12 Genomes study [21] were used for 11 of the species (*D*. *melanogaster*, *D*. *simulans*, *D*. *sechellia*, *D*. *yakuba*, *D*. *erecta*, *D*. *ananassae*, *D*. *pseudoobscura*, *D*. *persimilis*, *D*. *mojavensis*, *D*. *virilis*). The lines for the additional species were as follows: *D*. *mauritiana* (Dmau/[w1]; 14021–0241.60), *D*. *santomea* (STO-CAGO 1402–3; 14021–0271.01), *D*. *miranda* (MSH-22). Embryos were dechorionated, and imaged on a Zeiss Axioimager, under halocarbon oil, to determine stage. Since embryos were collected from a large number of mothers, it is unlikely that multiple samples came from the same mother. Stage 2 and late stage 5 embryos were identified based on morphology. Stage 2 embryos were selected based on the vitelline membrane retracting from both the anterior and posterior poles, prior to when pole cells become visible. Late stage 5 embryos were chosen based on having completed cellularization, but not yet having gastrulated. Embryos were then removed from the slide with a brush, cleaned of excess oil, placed into a drop of Trizol reagent (Ambion), and ruptured with a needle, then moved to a tube with more Trizol to be frozen at -80° C until extraction. RNA and DNA were extracted as in the manufacturer’s protocol, with the exception of extracting in an excess of reagent (1 mL was used) compared to expected mRNA and DNA concentration [26–28].

Extracted total RNA from single embryos was treated with the TurboDNA-free kit (Ambion) prior to library construction. Embryo mRNA-Seq libraries were generated for at least 3 replicate individuals per stage and per species, producing a total of 68 stage 2 and 76 stage 5 libraries, or 144 overall. More detail about sampling and replication is available in S14 Table. mRNA-Seq libraries were constructed using TruSeq RNA sample preparation kits (Illumina), using standard protocols, and indexed to pool 12 samples (embryos) per lane. Library concentration was measured using the Qubit fluorometer (Life Technologies) and the qPCR-based Library Quantification kits (KAPA biosystems), and size was measured using the Bioanalyzer (Agilent). Libraries were sequenced on an Illumina HiSeq 2000 DNA Sequencer.

mRNA-Seq libraries were constructed using poly(A) selection. This creates a potential source of bias, as poly(A)-tail length is highly regulated during oogenesis and early embryogenesis, especially for maternally deposited transcripts [10–12,14,15] However, there is previous evidence that the use of oligo(dT)-based poly(A) selection does not bias the transcripts recovered. A study [61] measuring both poly(A) tail length and transcript level during this period of development found that the dynamic changes in poly(A) tail length had minimal impact on the transcript abundance levels measured. To determine if use of oligo(dT)-based poly(A) selection may have biased the transcript level measurements in our experiment, we examined our mRNA-Seq data from *D*. *melanogaster* relative to two datasets of poly(A) tail length during early development in the same species [61,62]. In comparing the poly(A)-tail length of sequenced transcripts in our experiment to the total distribution of poly(A)-tail lengths each of these two experiments, we find no difference in distributions (Wilcox test, p = 0.74 compared to [61], p = 0.99 compared to [62]). While we cannot rule out that we are recovering a biased subset of transcripts due to oligo(dT) enrichment, it seems unlikely that this method produces a substantial bias.

### Genomes

Genome and annotation files for the 12 previously sequenced species [21], downloaded from Flybase [63], are listed in S15 Table. The *D*. *mauritiana* genome and assembly [22] were accessed from a website maintained by the Christian Schlötterer lab at the University of Veterinary Medicine Vienna. The *D*. *miranda* genome assembly (DroMir_2.2) [20,64,65] was downloaded from Pubmed and an annotation file was provided by the Doris Bachtrog lab at the University of California, Berkeley. A draft version of the *D*. *santomea* genome (using the non-inbred STO4 line), based on data from David Stern’s lab [66] was provided by Peter Andolfatto (Columbia University). We used an annotation of this genome, generated for us by Kevin Thornton (University of California, Irvine), to help construct the phylogeny of the 14 species (see “Phylogenetic analysis”, below). However, since the *D*. *santomea* genome was generated using non-inbred flies, we decided to map our *D*. *santomea* reads using the flybase *D*. *yakuba* assembly and annotation.

### Data processing

Reads were pre-processed using Cutadapt [67] to remove adapter contamination. Mapping and differential expression analysis were carried out using the Tuxedo suite [68], which allows for the discovery of novel isoforms and genes. Briefly, Tophat2, [69,70], which leverages Bowtie2 [71,72], was used to align reads to the reference and discover new splice junctions for each replicate of each stage and species. An assembly for each stage and replicate was generated using Cufflinks, where upper-quantile normalization was performed using the–N option, and all assemblies for each species were merged with CuffMerge. Using the merged assembly, FPKM levels were calculated with Cuffnorm, and differential expression between stages was assayed using CuffDiff.

With the aid of the output files from CuffDiff and CuffNorm, gene FPKM levels were calculated using the total of the FPKM levels for all isoforms of each gene. Assignment of orthologs relied on an orthology table from Flybase (“gene_orthologs_fb_2014_06_fixed.tsv”) and the *D*. *mauritiana* and *D*. *miranda* annotations described above. A table was generated (S16 Table) consisting of all genes with one-to-one orthologs in at least 12 of 14 species. If there was no known *D*. *melanogaster* ortholog for a gene in a given species, or multiple orthologs, that entry in the table was left blank and was not included in any of the analyses.

### Correlation analysis and clustering analysis

Spearman rank sum correlation coefficients were calculated using the R statistical environment [73]. When comparing species, only calculated FPKM values for genes with one-to-one orthologs were considered. Correlation plots and hierarchical clustering were generated using the R heatmap2 package.

### Comparisons with *Aedes aegypti*

Data from an *Aedes aegypti* transcriptomic time course [35] were compared to our *Drosophila* results. We used FPKM values from the 0–2 hour time period (the earliest available, and the one which is most likely to represent maternal RNA) and the 8–12 hour period (which corresponds to the completion of cellularization in *Aedes* [38], approximately equivalent to stage 5 in *Drosophila*) in our comparison. The data was gleaned from Supplemental S9 Table in Akbari et al. 2013. Using Inparanoid [74] we found a total of 4619 genes from this dataset that had one-to-one orthologs in *Drosophila melanogaster*. We expanded S16 Table to include orthologs from *Aedes*, using the data we had gathered from the Akbari et al., 2013 study.

### Phylogenetic analysis

The phylogeny was constructed using 21 loci, listed in S17 Table. These loci were selected from a previously published list [75] of 250 candidate genes for a *Drosphila* phylogenetic analysis (chosen based on low codon usage bias, availability of one-to-one orthologs, and other criteria). In selecting the loci for our study, we were limited by the quality of the available *D*. *santomea* genome and annotation, which was generated using the non-inbred STO4 line. The loci were aligned using MUSCLE, and regions of low quality in the alignment were removed using trimal. A total of 35,829 base pairs were used in generation of the phylogeny, of which 19,176 were informative.

MrBayes3.2 was used to generate the phylogeny and infer ancestral stage 2 and stage 5 states for 8,075 genes with orthologs in 12 of the 14 species. Each gene was assigned a binary of state of 1 (present) if the FPKM level at a given stage was greater than or equal to the chosen threshold and 0 if it was below the threshold. If there was no one-to-one ortholog in a species, the state was considered unknown. Following previous transcriptomic analyses [35], an FPKM threshold of 1 was selected. Our dataset thus consisted of 16,150 states (two per species per gene) in addition to the 35,829 nucleotides of DNA. For the binary data, the frequency of state 1 was 0.715, the frequency of state 0 was 0.219, and the frequency of unknown states was 0.0656. MrBayes was run separately 12 times to reconstruct ancestral states on each internal node of the phylogeny (a MrBayes file for reconstructing the ancestral state for the *obscura* group is found in S1 Table). For the nucleotide data, we used a GTR model and a gamma distribution to model rate variation across sites. Each chain was run for 200,000 generations with a burn-in fraction of 0.25.

To study changes along the phylogeny, a gain in representation of a gene at a given stage was recorded if an inferred state changed from 0 (with at least 90% posterior probability) in the ancestral node to 1 (was at least 90% posterior probability) in the derived node, while a change from 1 to 0 (with 90% posterior probability in each case) was designated as a loss.

### Unannotated gene analysis

Unannotated genes were categorized as those given numbers by the Tuxedo suite but not found in the reference genome annotations (download from flybase or another source, as described above) that we provided to the software pipeline.

### Stage-specific isoform analysis

Isoforms were classified as maternal (M) if they were present at stage 2 and absent (below the FPKM threshold of 1) at stage 5, while they were considered zygotic (Z) if they were only present at stage 5. Isoforms that were present at both stages were filtered to select those that showed significant differences between stages (q value less than 0.05 in the CuffDiff output). From this set, those where the level at one stage was at least twice that of the other stage were categorized as primarily maternal (PM) if stage 2 was higher or primarily zygotic (if stage 5) was higher. All other isoforms that were present at the two stages were classified as maternal-zygotic (MZ).

Genes where at least one isoform was primarily maternal and the other was primarily zygotic show evidence of stage-specific isoform usage and were given the ALT classification. Using custom Perl scripts, these genes were identified and the extent of the conservation of the ALT state (across the 14 species) was assessed.

## Supporting information

S1 Fig Clustering demonstrates within- developmental stage similarity across *Drosophila*. A heatmap comparing all 28 transcriptomes shows that transcriptomes of each stage cluster together. While closely related species generally cluster with each other for both stage 2 and stage 5, the heatmap does not fully recapitulate the phylogeny. In both cases, transcriptomes from the *obscura* group form an outgroup to those from all other species of an equivalent stage. This finding is true when examining all transcripts (panel A), autosomal transcripts only (panel B), or autosomal transcripts only when *A*. *aegypti* is included (panel C, i.e. the autosomal-only version of Fig 4).
(PDF)

S2 Fig The number of genes that are maternal-only and zygotic-only in both species when comparing *D. melanogaster* to the other 13 species. The number of common zygotic-only genes decreases at evolutionary distances greater than 10 million years (i.e. when the second species is outside of the *melanogaster* subgroup), but remains close to 450 at distances of close to 60 million years. In contrast, the number of common maternal-only genes is much lower and rapidly declines to less than 100.
(PDF)

S3 Fig Patterns of transcriptomic evolution across different classes of genes. Separate correlation plots show interspecific pairwise Spearman correlation coefficients of transcript levels (FPKM) when considering all genes represented at stage 2 and stage 5 in at least one of the two species (top row) and genes that are maternal-only (represented at stage 2 with all transcripts degraded by stage 5) or zygotic-only (represented at stage 5 and not maternally deposited) in at least one of the species in a pair (bottom row). Compare with Fig 3 in the main text, in which included genes need to share gene category (ex. maternal-only) in both species. The difference is most striking for the zygotic-only genes (bottom right in both figures), which show much lower correlation coefficients when all genes that are zygotic-only in either of the two species are compared.
(PDF)

S4 Fig A core set of developmental genes are zygotic-only in both *Drosophila* and *Aedes*. (A) A gene that is zygotic-only in both *Aedes aegypti* and at least one *Drosophila* species is likely to be zygotic-only in other *Drosophila* species as well. (B) If the gene is zygotic-only in *Aedes aegypti*, *D*. *willistoni* (the most basal species in the *Sophophora* clade) and at least one of the species in the *Drosophila* subgenus (i.e. *D*. *virilis* and *D*. *mojavensis*), it has a strong probability of being zygotic-only in the vast majority of *Drosophila* species. (C,D) Word clouds showing the GO enrichment terms for “molecular function” (C) and the top 30 terms for “biological process” (D) when the conserved set of genes described in (B) is compared to the set of all genes with transcripts at stage 5. In both word clouds, the size of the word is proportional to the fold enrichment.
(PDF)

S5 Fig GO enrichment for stage 5 gains. This word cloud shows the GO term enrichment for transcripts that show a gain in stage 5 representation in any lineage, when compared to all genes represented at stage 5. The size of the word is proportional to the fold enrichment. See S8 Table for more information.
(PDF)

S6 Fig Genes uniquely represented in each species at each of the two stages. For all genes with one-to-one orthologs in at least 12 of the 14 species, we show how many genes are above a higher threshold of FPKM = 3 in one species and below a lower threshold of FPKM = 1 in all other species. Generally, there are more transcripts with species-specific representation at stage 2 than stage 5, and *D*. *virilis* has exceptionally high numbers of species-specific transcripts at both stages.
(PDF)

S7 Fig Properties of unannotated genes. These genes, as described in the text, are unannotated, and a majority are taxonomically restricted. Genes are divided into the following categories: maternal-only (M), predominantly maternal (PM), predominantly zygotic (PZ), and zygotic-only (Z); see results for further description. A) Unannotated genes have smaller numbers of exons for all categories of genes as compared to all genes. Comparing species means for exon number between the maternal (M, PM) and zygotic (PZ, Z) categories, the maternal categories have significantly higher exon numbers than zygotic genes for both the set of all genes and the set of unannotated genes (t-test, p<0.01). B) Unannotated genes have shorter introns and exons than the set of all genes. Both all genes and unannotated genes have no significant differences in exon length between the maternal and zygotic classes of genes. However, both the set of all genes and the unannotated genes have significantly longer introns at the maternal stages (t-test, p<0.0001).
(PDF)

S8 Fig Properties of genes with stage specific or stage restricted isoforms. These genes, referred to as “ALT” genes, as described in the text, are those with isoforms present or predominant at one developmental stage, and a different isoform present or predominant at the other. Genes are divided into the following categories: maternal-only (M), predominantly maternal (PM), predominantly zygotic (PZ), and zygotic-only (Z); see results for further description. A) ALT genes have larger numbers of exons for all categories of genes as compared to all genes. Comparing species means for exon number between the maternal (M, PM) and zygotic (PZ, Z) categories, the maternal categories have significantly higher exon numbers than zygotic genes for all genes only (Wilcoxon test, p = 9.17x10^-6^), while ALT genes do not have significant differences in exon number between stages (Wilcoxon test, p = 0.43. B) ALT genes have slightly longer exons and much larger introns than the set of all genes. The set of all genes has no significant difference in the length of exons between the maternal and zygotic isoforms (Wilcoxon test, p = 0.18). Surprisingly, the ALT genes have a small but significant (Wilcoxon test, p = 0.0008) difference between maternal and zygotic isoforms for exon length, with zygotic isoforms being slightly longer. Both the set of all genes (Wilcoxon test, p = 4.99x10^-8^) and the ALT genes (Wilcoxon test, p = 0.0001) have significantly longer introns at the maternal stages.
(PDF)

S1 Table Spearman correlation coefficients between replicate FPKM levels, organized by species. Stage 5 samples, in which the sex has been determined, are labeled male (M) or female (F).
(XLSX)

S2 Table Spearman correlations coefficients for comparisons between all species, over all gene categories. Tabs are labeled by gene category (stage 2, stage 5, maternal-only, zygotic-only) and transcript selection criteria. Transcripts were selected for comparison in two different ways: either both species being compared had to share the stage definition (e.g. transcript is zygotic only in both species; this is denoted by “present in both” on the tab), or only one species was required to have a gene meet this definition (e.g. transcript is zygotic-only in one species, it is included in the zygotic-only correlations; denoted by “at least one” on the tab). The first method, where both species were required to share the gene category for a gene to be included in the calculation is the method shown in all main text figures.
(XLSX)

S3 Table Number and proportion of stage 2-only and stage 5-only genes in each of the 14 species. We found 173 genes where the stage 5-only state was conserved between *Aedes* and one of the *Drosophila* species, and identified a core set of 61 where this state was highly conserved.
(XLSX)

S4 Table A list of all genes that are zygotic-only in *Aedes aegypti* and at least one of the 14 Drosophila species, with mean stage 2 and stage 5 levels for each species. The final column shows the total number of species (including *A*. *aegypti*) in which the gene is zygotic-only.
(XLSX)

S5 Table A list of genes which are zygotic-only in *Aedes aegypti* and at least two *Drosophila* species from the two most basal clades in our study (D. virilis or D. mojavensis from the Drosophila subgenus and D. willistoni from New World Sophophora). Mean stage 2 and stage 5 levels in for each species are shown.
(XLSX)

S6 Table A table showing gains and losses at relevant nodes on the phylogeny. Genes were assigned a value of “0” at a given stage for a given species if the FPKM level was <1 and “1” if the FPKM level was > = 1. All cases where the gene was assigned one value in the outgroup (at > = 90% posterior probability) and a different value in the ingroup at > = 90% posterior probability, are shown. The chromosome or scaffold on which the gene is located, and stage 2 and stage 5 levels, are shown for each species.
(XLSX)

S7 Table Evolutionary transitions. All stage 2 and stage 5 transitions where a binary state (0 = absent, 1 = present) was identified with 90% posterior probability in the outgroup clade, amd a different binary state was identified at 90% posterior probability in the ingroup clade, are listed.
(TXT)

S8 Table A gene ontology analysis using DAVID comparing the combined set of all genes showing a stage 5 gain (at any node on the phylogeny) to all genes represented at stage 5. The analysis considered all three GO term categories: molecular function (MF), cellular component (CC) and biological process (BP).
(XLSX)

S9 Table A table showing genes uniquely represented at each of the two stages. For all genes with one-to-one orthologs in at least 12 of the 14 species, the number of genes that were represented at a given stage in only one species, with a threshold of FPKM = 1, is shown. To eliminate cases where a gene is only slightly above the threshold in one species, we also show how many genes are above a higher threshold of FPKM = 3 in one species and below a lower threshold of FPKM = 1 in all other species. Note that at stage 2, *D*. *erecta* and *D*.*virilis* show the largest number of genes that are uniquely represented, while *D*. *virilis* has more than twice as many genes with FPKM > 3 where all one-to-one orthologs are not represented (FPKM < 1) in all other species.
(XLSX)

S10 Table A table showing basic gene and isoform statistics for different categories of genes across the 14 species, including all genes, genes with alternative isoforms (ALT), unannotated genes (UNAN) and unannotated genes with alternative isoforms (ALT UNAN). Mean exon number and mean exonic and intronic lengths are shown for each category of gene. Data is shown for isoforms that are maternal-only (M), predominantly maternal (PM), maternal and zygotic (MZ), predominantly zygotic (PZ) and zygotic-only (Z). See the main text for an explanation of these terms.
(XLSX)

S11 Table Genes with alternative isoforms, with gene summaries from flybase.org. The table shows all genes where at least one ortholog has an isoform that is maternal or predominantly maternal, and an alternative isoform that is zygotic or predominantly zygotic (labeled “ALT”). In each case where this occurs, all one-to-one orthologs in other species and their associated isoforms are shown. For each isoform, FPKM levels for stage 2 and stage 5, the number of exons, the total exonic length, and Cufflinks transcript classification (http://cole-trapnell-lab.github.io/cufflinks/cuffcompare/), respectively, are recorded, and the isoform is labeled M (maternal-only), PM (predominantly maternal), MZ (maternal and zygotic), PZ (predominantly zygotic) or Z (zygotic-only). See the main text for an explanation of these terms.
(XLSX)

S12 Table DAVID gene ontology analysis of ALT genes when compared to all genes represented at stage 2 or stage 5. (XLSX)

S13 Table Alternative isoform statistics. There are a total of 3,430 genes where at least one ortholog is maternal or predominantly maternal at one stage and zygotic or predominantly zygotic at the other stage (ALT pattern). For 129 genes, the ALT state is conserved in orthologs from at least 7 species.
(XLSX)

S14 Table Sample information. The number of replicates for each stage of each species are shown. References for previously published data are included.
(XLSX)

S15 Table List of genome and annotation files used in this analysis. (DOCX)

S16 Table Gene expression across the 14 *Drosophila* species and *Aedes aegypti*, with each row containing a set of orthologs. The gene name, followed by the stage 2 and stage 5 levels, are included. An empty cell indicates that a one-to-one ortholog was not identified.
(XLSX)

S17 Table List of loci used in the phylogenetic analysis. (DOCX)

S18 Table The MrBayes file used in the phylogenetic analysis for reconstruction of ancestral states on the *obscura* group node. The MrBayes files used for ancestral state reconstruction on the other nodes of the phylogeny were identical to this one, with the exception of lines 54 and 55 where the node is defined.
(NEX)
