# Supplementary figures and images for: Evolution of maternal and zygotic mRNA complements in the early Drosophila embryo
Source: PLoS Genet. 2018 Dec 17;14(12):e1007838. doi: 10.1371/journal.pgen.1007838 (PMC6312346; doi:10.1371/journal.pgen.1007838)

A

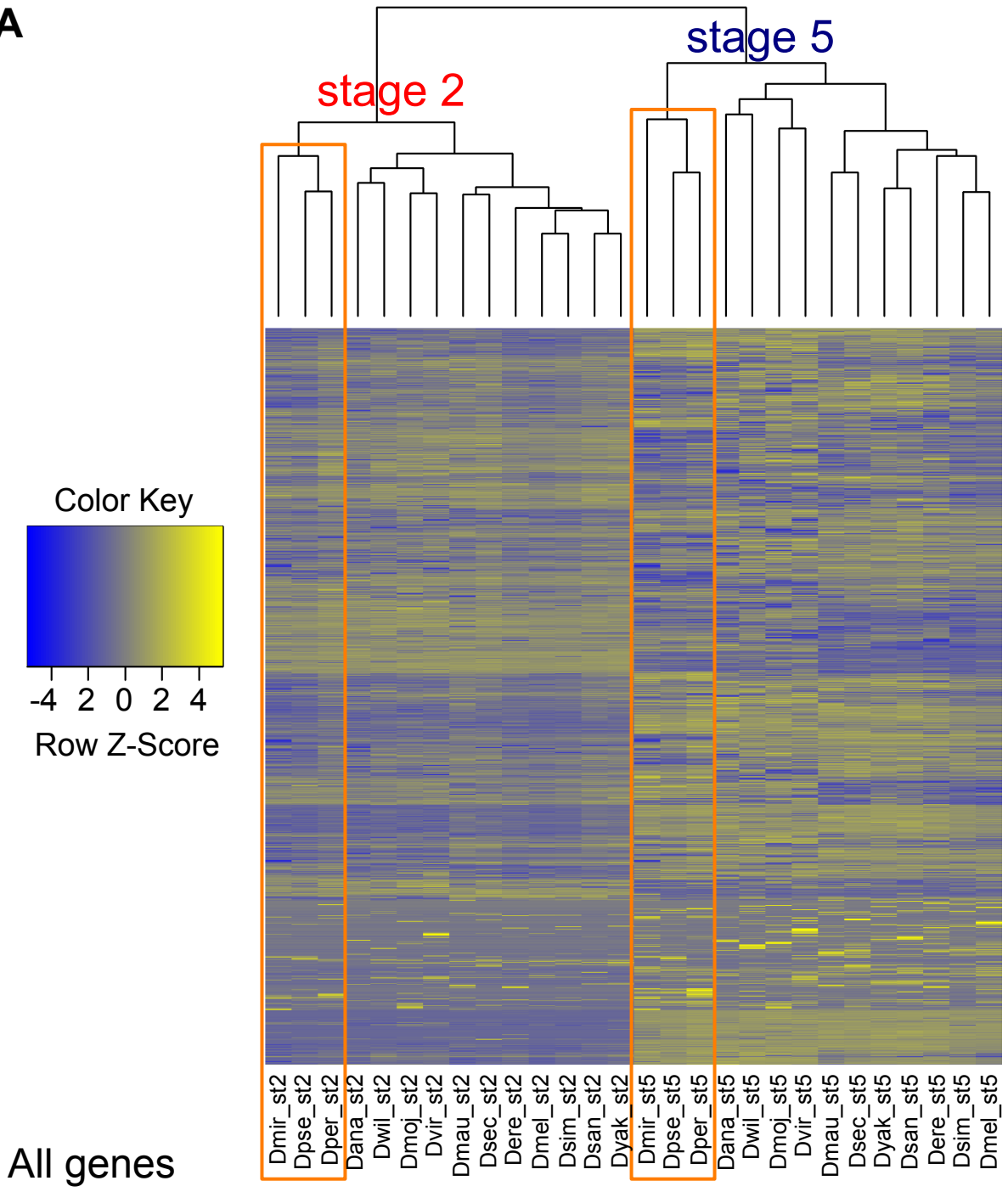

**B**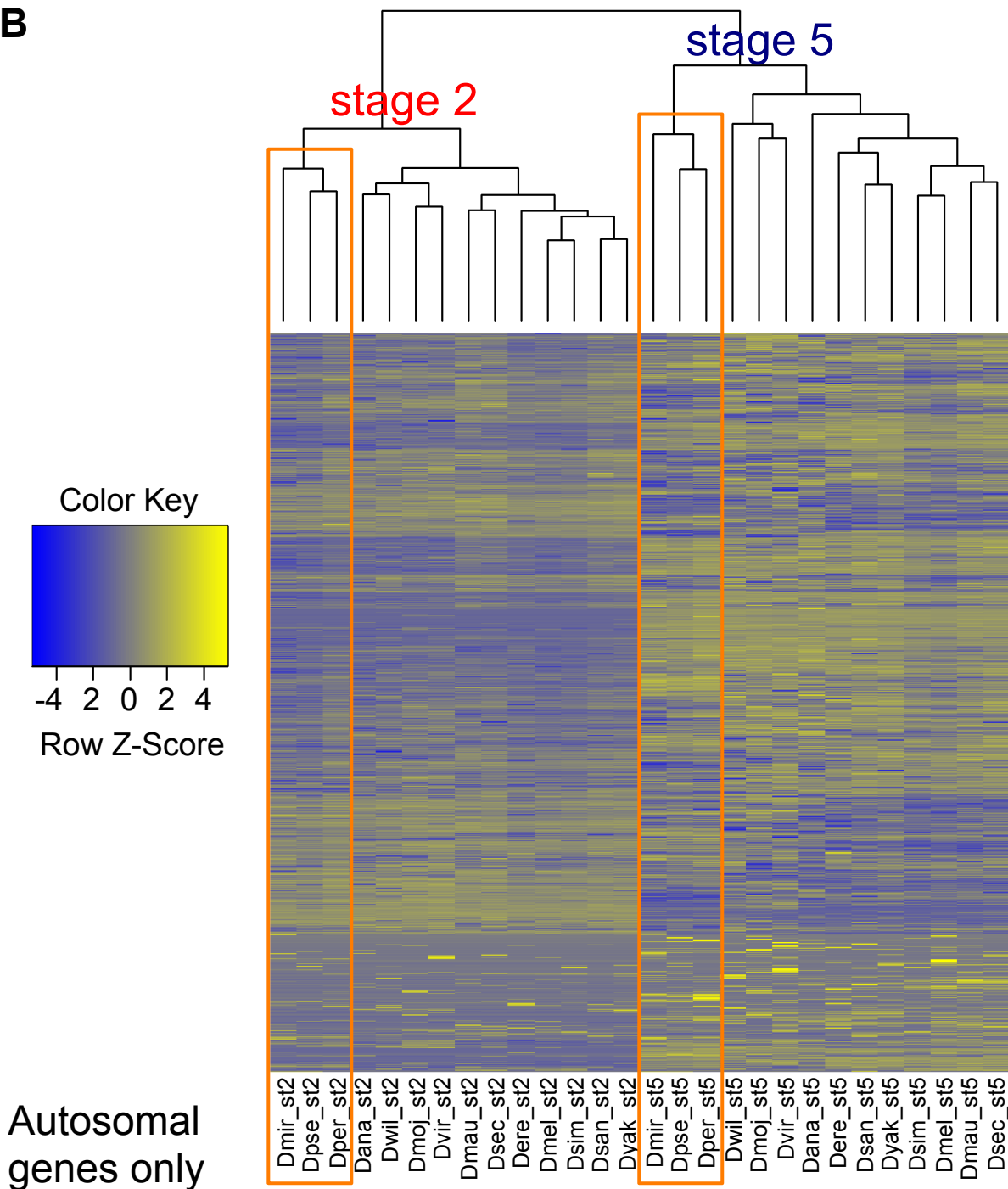

C

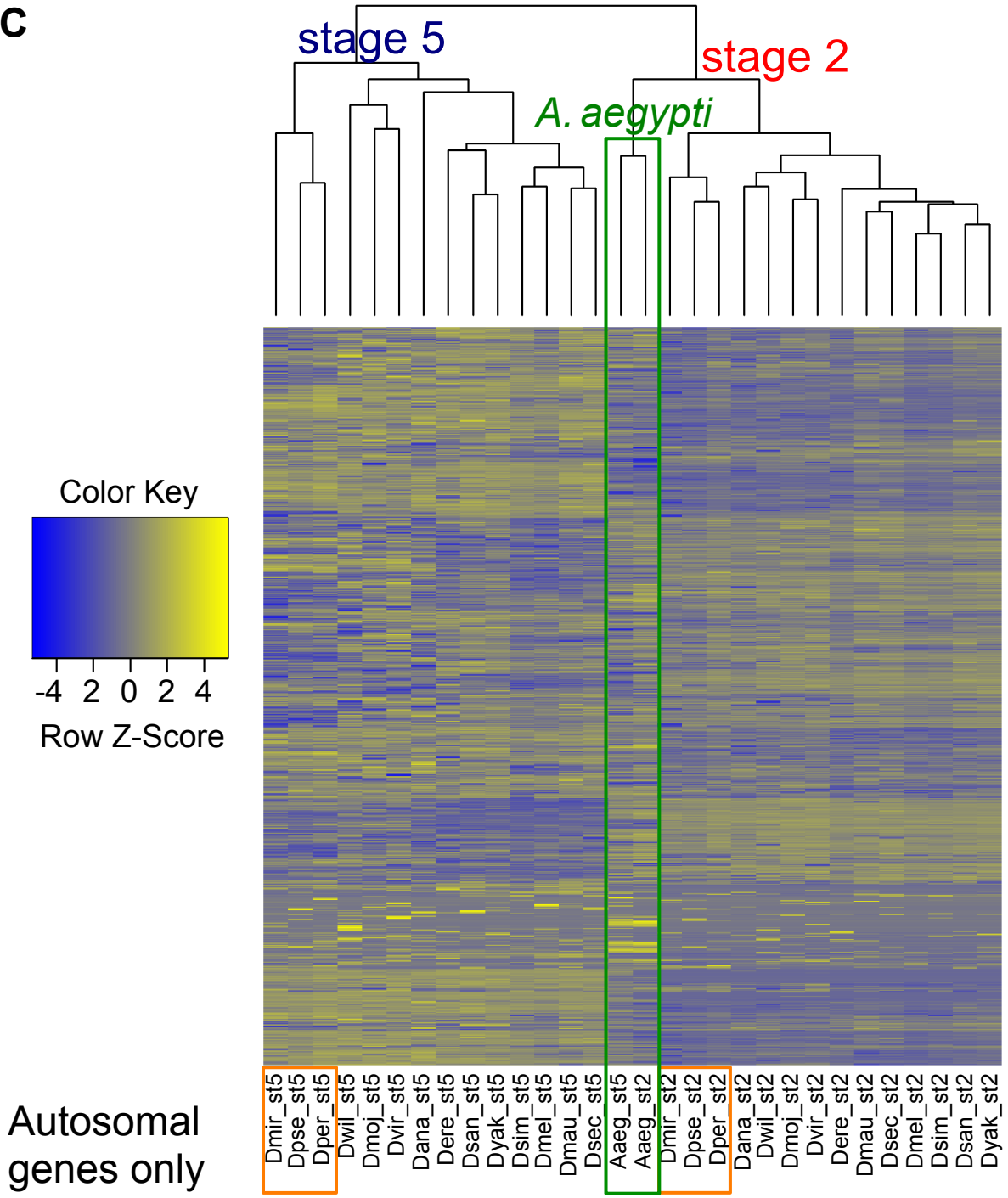

Supplement: S1 Fig — A heatmap comparing all 28 transcriptomes shows that transcriptomes of each stage cluster together. While closely related species generally cluster with each other for both stage 2 and stage 5, the heatmap does not fully recapitulate the phylogeny. In both cases, transcriptomes from the obscura group form an outgroup to those from all other species of an equivalent stage. This finding is true when examining all transcripts (panel A), autosomal transcripts only (panel B), or autosomal transcripts only when A. aegypti is included (panel C, i.e. the autosomal-only version of Fig 4). (PDF) [file pgen.1007838.s001.pdf]

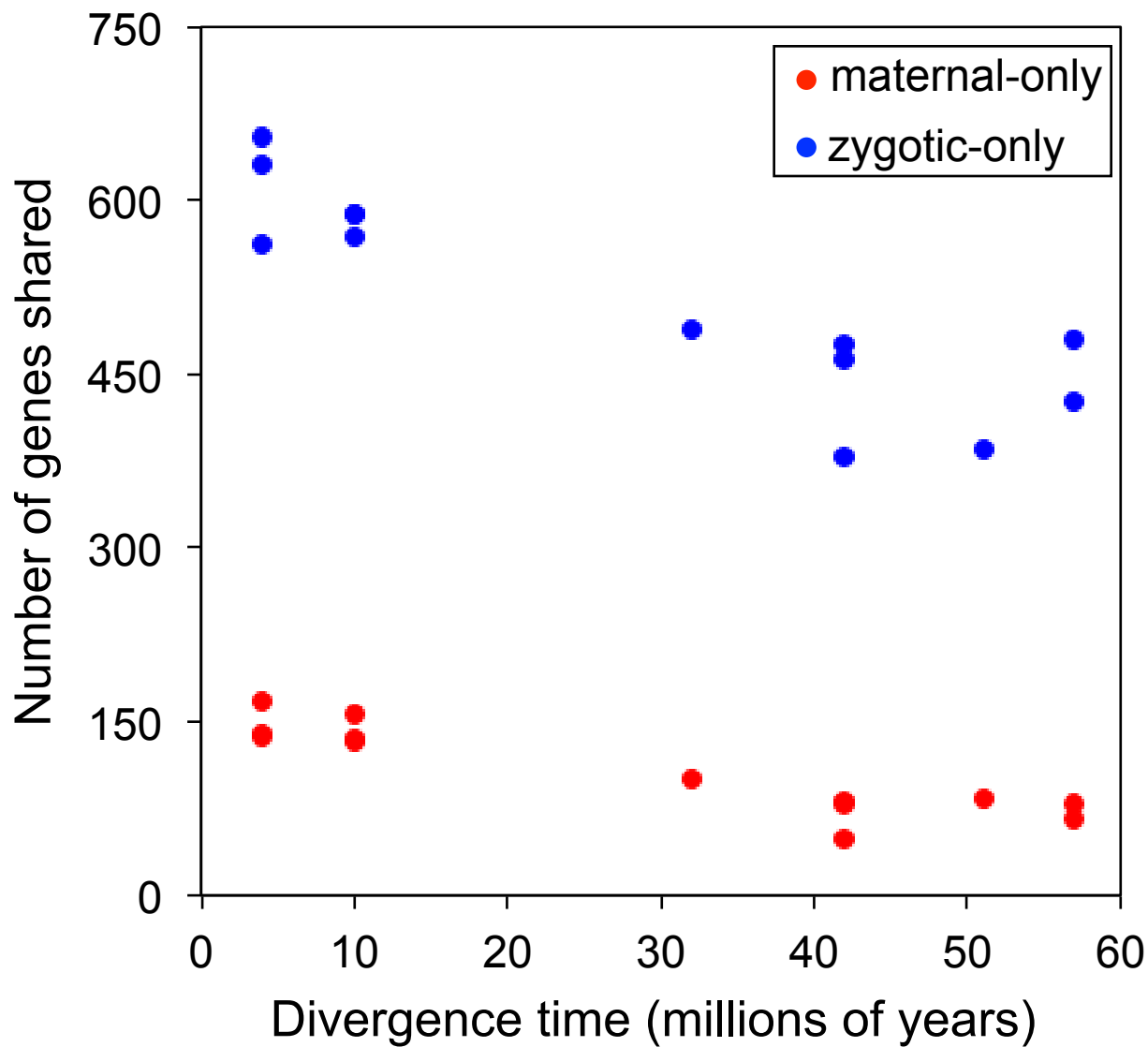

Supplement: S2 Fig — The number of common zygotic-only genes decreases at evolutionary distances greater than 10 million years (i.e. when the second species is outside of the melanogaster subgroup), but remains close to 450 at distances of close to 60 million years. In contrast, the number of common maternal-only genes is much lower and rapidly declines to less than 100. (PDF) [file pgen.1007838.s002.pdf]

all genes:

stage 2

stage 5

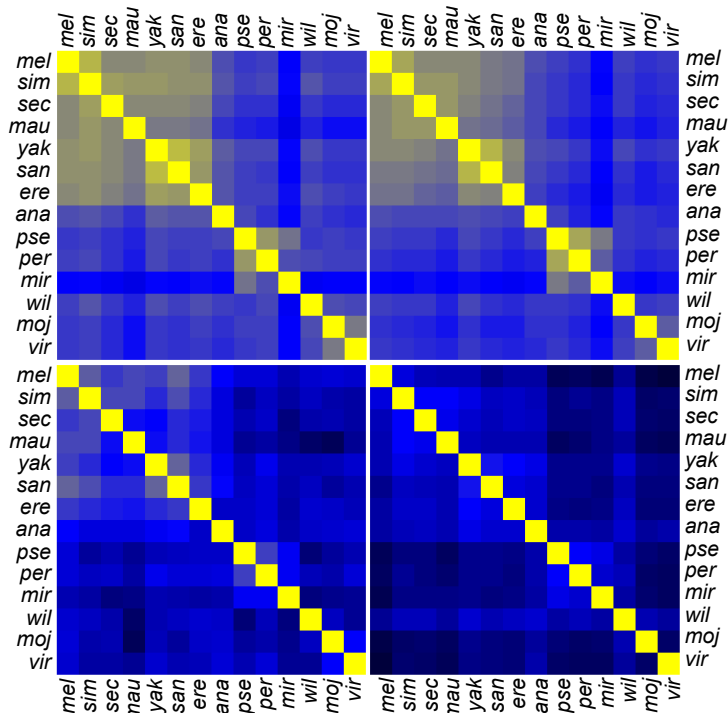

Color Key

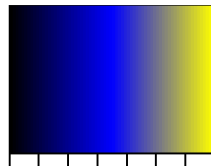

0.3 0.5 0.7 0.9

Spearman's  
correlation

stage-  
restricted:

stage 2 only

stage 5 only

Supplement: S3 Fig — Separate correlation plots show interspecific pairwise Spearman correlation coefficients of transcript levels (FPKM) when considering all genes represented at stage 2 and stage 5 in at least one of the two species (top row) and genes that are maternal-only (represented at stage 2 with all transcripts degraded by stage 5) or zygotic-only (represented at stage 5 and not maternally deposited) in at least one of the species in a pair (bottom row). Compare with Fig 3 in the main text, in which included genes need to share gene category (ex. maternal-only) in both species. The difference is most striking for the zygotic-only genes (bottom right in both figures), which show much lower correlation coefficients when all genes that are zygotic-only in either of the two species are compared. (PDF) [file pgen.1007838.s003.pdf]

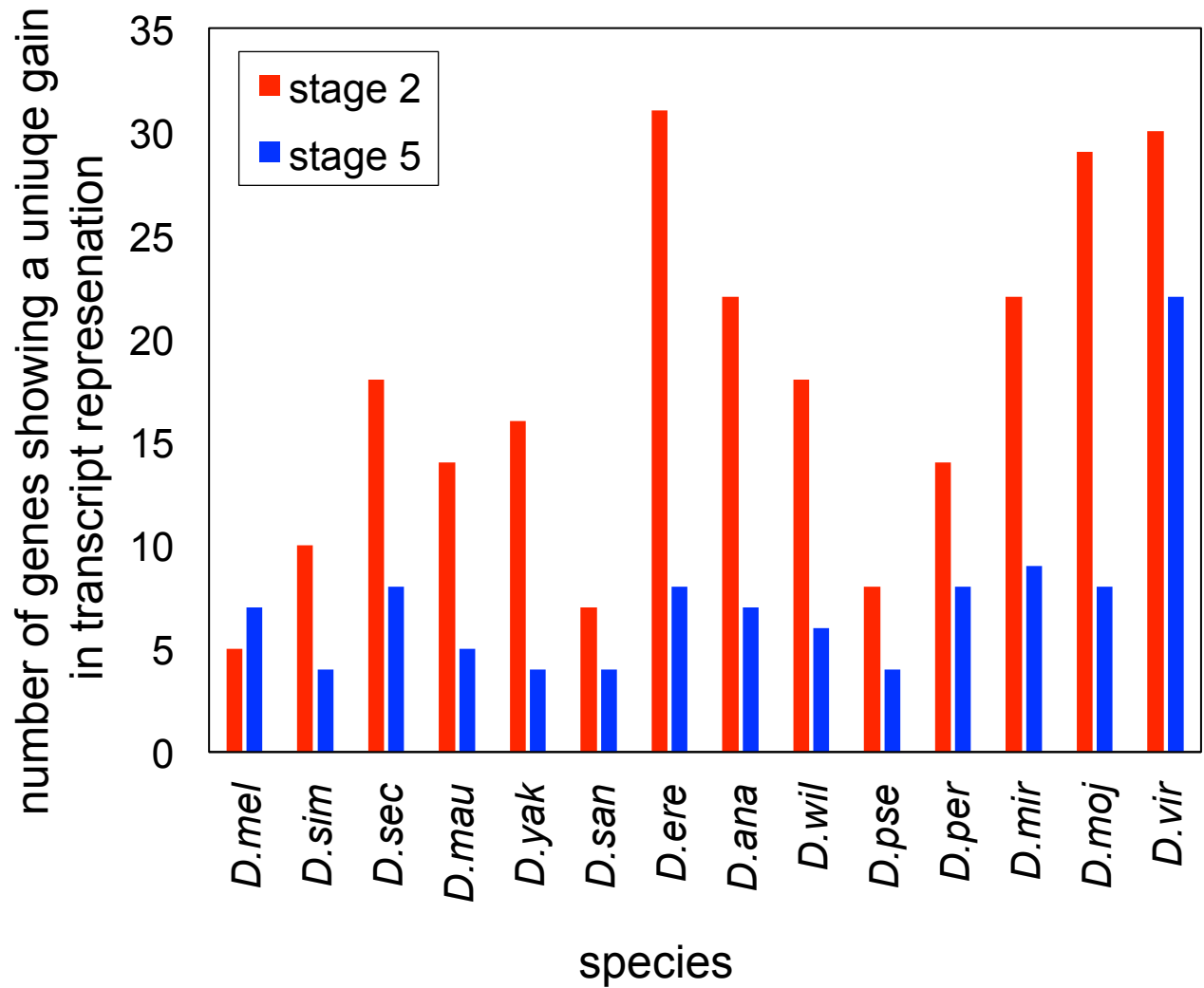

Supplement: S6 Fig — For all genes with one-to-one orthologs in at least 12 of the 14 species, we show how many genes are above a higher threshold of FPKM = 3 in one species and below a lower threshold of FPKM = 1 in all other species. Generally, there are more transcripts with species-specific representation at stage 2 than stage 5, and D. virilis has exceptionally high numbers of species-specific transcripts at both stages. (PDF) [file pgen.1007838.s006.pdf]

# Properties of unannotated genes

**A**

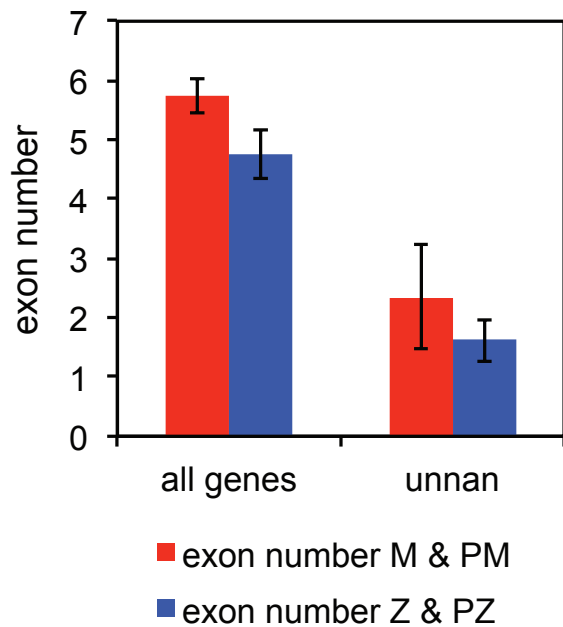

**B**

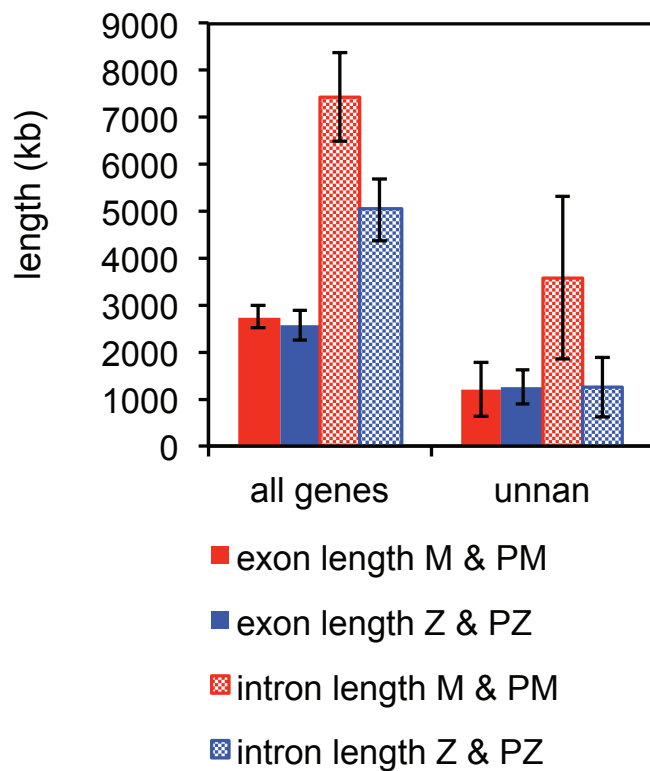

Supplement: S7 Fig — These genes, as described in the text, are unannotated, and a majority are taxonomically restricted. Genes are divided into the following categories: maternal-only (M), predominantly maternal (PM), predominantly zygotic (PZ), and zygotic-only (Z); see results for further description. A) Unannotated genes have smaller numbers of exons for all categories of genes as compared to all genes. Comparing species means for exon number between the maternal (M, PM) and zygotic (PZ, Z) categories, the maternal categories have significantly higher exon numbers than zygotic genes for both the set of all genes and the set of unannotated genes (t-test, p<0.01). B) Unannotated genes have shorter introns and exons than the set of all genes. Both all genes and unannotated genes have no significant differences in exon length between the maternal and zygotic classes of genes. However, both the set of all genes and the unannotated genes have significantly longer introns at the maternal stages (t-test, p<0.0001). (PDF) [file pgen.1007838.s007.pdf]

# Properties of genes with stage-specific or stage-predominant isoforms

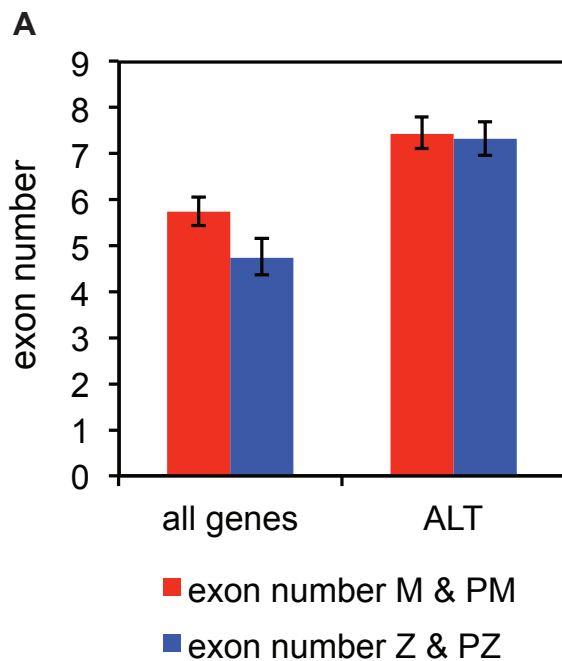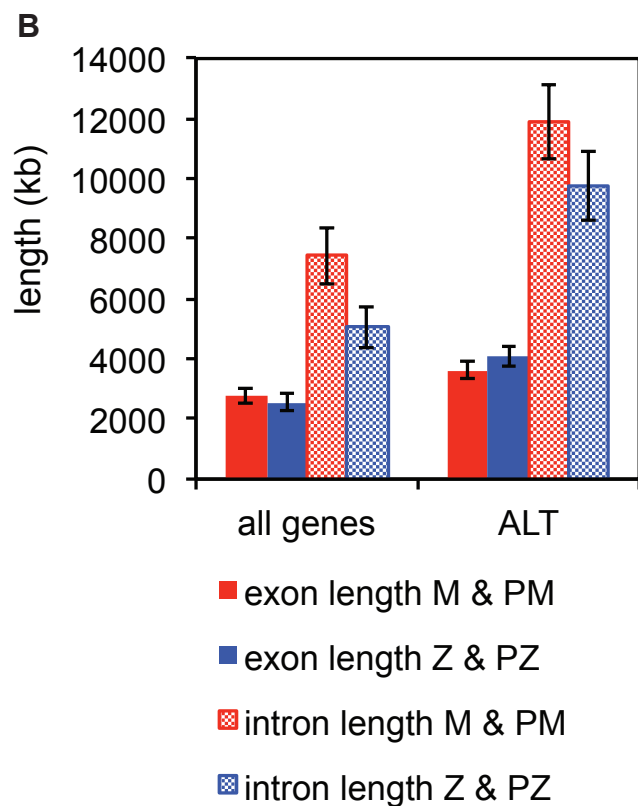

Supplement: S8 Fig — These genes, referred to as “ALT” genes, as described in the text, are those with isoforms present or predominant at one developmental stage, and a different isoform present or predominant at the other. Genes are divided into the following categories: maternal-only (M), predominantly maternal (PM), predominantly zygotic (PZ), and zygotic-only (Z); see results for further description. A) ALT genes have larger numbers of exons for all categories of genes as compared to all genes. Comparing species means for exon number between the maternal (M, PM) and zygotic (PZ, Z) categories, the maternal categories have significantly higher exon numbers than zygotic genes for all genes only (Wilcoxon test, p = 9.17x10^-6^), while ALT genes do not have significant differences in exon number between stages (Wilcoxon test, p = 0.43. B) ALT genes have slightly longer exons and much larger introns than the set of all genes. The set of all genes has no significant difference in the length of exons between the maternal and zygotic isoforms (Wilcoxon test, p = 0.18). Surprisingly, the ALT genes have a small but significant (Wilcoxon test, p = 0.0008) difference between maternal and zygotic isoforms for exon length, with zygotic isoforms being slightly longer. Both the set of all genes (Wilcoxon test, p = 4.99x10^-8^) and the ALT genes (Wilcoxon test, p = 0.0001) have significantly longer introns at the maternal stages. (PDF) [file pgen.1007838.s008.pdf]
