# Supplementary material for: Evolution of maternal and zygotic mRNA complements in the early Drosophila embryo
Source: PLoS Genet. 2018 Dec 17;14(12):e1007838. doi: 10.1371/journal.pgen.1007838 (PMC6312346; doi:10.1371/journal.pgen.1007838)
Supplement: S4 Fig — (A) A gene that is zygotic-only in both Aedes aegypti and at least one Drosophila species is likely to be zygotic-only in other Drosophila species as well. (B) If the gene is zygotic-only in Aedes aegypti, D. willistoni (the most basal species in the Sophophora clade) and at least one of the species in the Drosophila subgenus (i.e. D. virilis and D. mojavensis), it has a strong probability of being zygotic-only in the vast majority of Drosophila species. (C,D) Word clouds showing the GO enrichment terms for “molecular function” (C) and the top 30 terms for “biological process” (D) when the conserved set of genes described in (B) is compared to the set of all genes with transcripts at stage 5. In both word clouds, the size of the word is proportional to the fold enrichment. (PDF) [file pgen.1007838.s004.pdf]

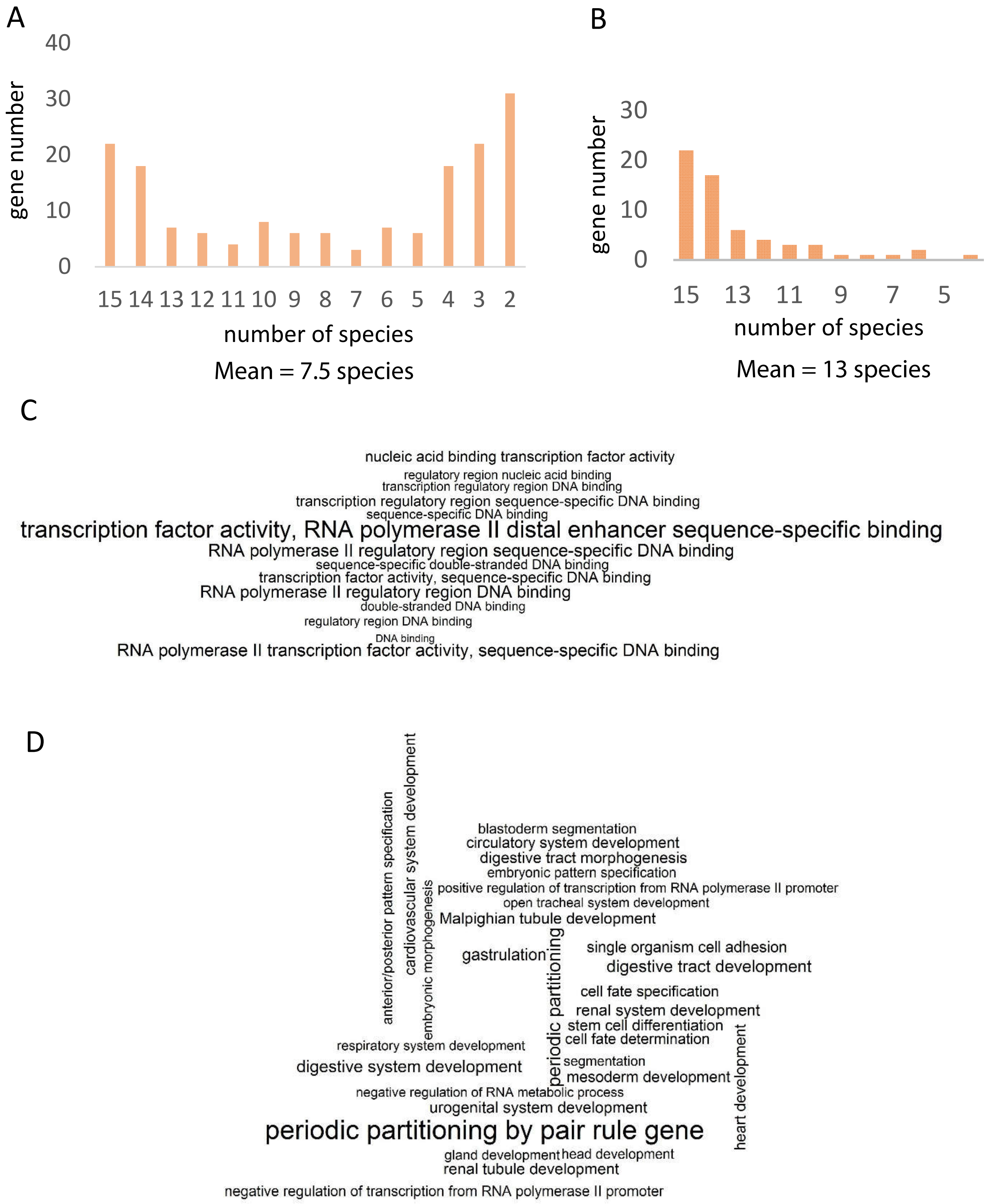
A
gene number
40
30
20
10
0
15 14 13 12 11 10 9 8 7 6 5 4 3 2
number of species
Mean = 7.5 species
B
gene number
30
20
10
0
15 13 11 9 7 5
number of species
Mean = 13 species
C
nucleic acid binding transcription factor activity
regulatory region nucleic acid binding
transcription regulatory region DNA binding
transcription regulatory region sequence-specific DNA binding
sequence-specific DNA binding
transcription factor activity, RNA polymerase II distal enhancer sequence-specific binding
RNA polymerase II regulatory region sequence-specific DNA binding
sequence-specific double-stranded DNA binding
transcription factor activity, sequence-specific DNA binding
RNA polymerase II regulatory region DNA binding
double-stranded DNA binding
regulatory region DNA binding
DNA binding
RNA polymerase II transcription factor activity, sequence-specific DNA binding
D
anterior/posterior pattern specification
cardiovascular system development
embryonic morphogenesis
blastoderm segmentation
circulatory system development
digestive tract morphogenesis
embryonic pattern specification
positive regulation of transcription from RNA polymerase II promoter
open tracheal system development
Malpighian tubule development
gastrulation
periodic partitioning
single organism cell adhesion
digestive tract development
cell fate specification
renal system development
stem cell differentiation
cell fate determination
respiratory system development
digestive system development
segmentation
mesoderm development
heart development
negative regulation of RNA metabolic process
urogenital system development
periodic partitioning by pair rule gene
gland development
head development
renal tubule development
negative regulation of transcription from RNA polymerase II promoter
