# Supplementary material for: Evolution of maternal and zygotic mRNA complements in the early Drosophila embryo
Source: PLoS Genet. 2018 Dec 17;14(12):e1007838. doi: 10.1371/journal.pgen.1007838 (PMC6312346; doi:10.1371/journal.pgen.1007838)

GO enrichment for the genes that show a gain in stage 5 representation during evolution (in any lineage) when compared to all genes represented at stage 5

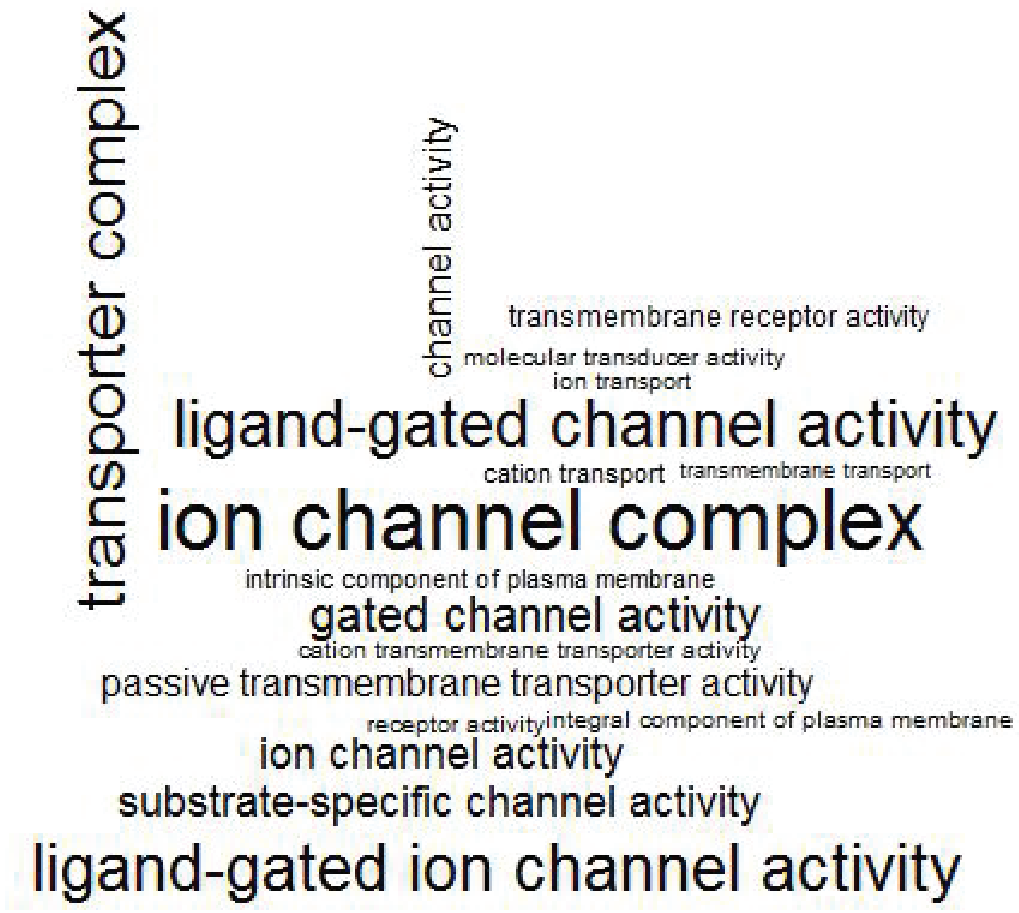

Supplement: S5 Fig — This word cloud shows the GO term enrichment for transcripts that show a gain in stage 5 representation in any lineage, when compared to all genes represented at stage 5. The size of the word is proportional to the fold enrichment. See S8 Table for more information. (PDF) [file pgen.1007838.s005.pdf]
