# Supplementary material for: Evolution of maternal and zygotic mRNA complements in the early Drosophila embryo
Source: PLoS Genet. 2018 Dec 17;14(12):e1007838. doi: 10.1371/journal.pgen.1007838 (PMC6312346; doi:10.1371/journal.pgen.1007838)
Supplement: S15 Table — (DOCX) [file pgen.1007838.s023.docx]

**Genome Files**

dana-all-chromosome-r1.3.fasta

dere-all-chromosome-r1.3.fasta

Dmau_MS17_all_chromosomes_r.1.0.fa

dmel-all-chromosome-r6.02.fa

Dmir.V2.2_Muller.fa

dmoj-all-chromosome-r1.3.fa

dper-all-chromosome-r1.3.fa

dpse-all-chromosome-r3.2.fa

dsec-all-chromosome-r1.3.fa

dsim-all-chromosome-r2.01.fa

dvir-all-chromosome-r1.2.fa

dwil-all-chromosome-r1.3.fa

dyak-all-chromosome-r1.3.fa

**Annotation Files**

dana-all-r1.3.gff

dere-all-r1.3.gff

Dmau_annotation_5_sets_edited.gff3

dmel-all-filtered-r6.02.gff

Dmir.makergene.gff3

dmoj-all-r1.3.gff

dper-all-r1.3.gff

dpse-all-r3.2.gff

dsec-all-r1.3.gff

dsim-all-r2.01.gff

dvir-all-r1.2.gff

dwil-all-r1.3.gff

dyak-all-r1.3.gff
