# Supplementary material for: Evolution of maternal and zygotic mRNA complements in the early Drosophila embryo
Source: PLoS Genet. 2018 Dec 17;14(12):e1007838. doi: 10.1371/journal.pgen.1007838 (PMC6312346; doi:10.1371/journal.pgen.1007838)
Supplement: S17 Table — (DOCX) [file pgen.1007838.s025.docx]

List of loci used in the phylogenetic analysis

FBgn0034962

FBgn0031881

FBgn0033557

FBgn0032258

FBgn0033055

FBgn0032670

FBgn0027565

FBgn0036036

FBgn0034649

FBgn0026371

FBgn0039158

FBgn0032881

FBgn0032250

FBgn0034442

FBgn0037743

FBgn0037661

FBgn0042110

FBgn0035166

FBgn0033615

FBgn0040273

FBgn0033672
